# Comparative *O*-GlcNAc Proteomic Analysis Reveals a Role of *O*-GlcNAcylated SAM68 in Lung Cancer Aggressiveness

**DOI:** 10.3390/cancers14010243

**Published:** 2022-01-04

**Authors:** Chia-Hung Lin, Chen-Chung Liao, Shu-Ying Wang, Chia-Yi Peng, Yi-Chen Yeh, Mei-Yu Chen, Teh-Ying Chou

**Affiliations:** 1 Department of Pathology and Laboratory Medicine, Taipei Veterans General Hospital, Taipei 11227, Taiwan; chiahunglin0222@gmail.com (C.-H.L.); ycyeh2@vghtpe.gov.tw (Y.-C.Y.); 2 Metabolomics-Proteomics Research Center, National Yang Ming Chiao Tung University, Taipei 11221, Taiwan; ccliao@nycu.edu.tw; 3 Institute of Clinical Medicine, College of Medicine, National Yang Ming Chiao Tung University, Taipei 11221, Taiwan; sywang15@vghtpe.gov.tw; 4 Institute of Biochemistry and Molecular Biology, College of Life Sciences, National Yang Ming Chiao Tung University, Taipei 11221, Taiwan; ivy110024@gmail.com; 5 Faculty of Medicine, School of Medicine, National Yang Ming Chiao Tung University, Taipei 11221, Taiwan; 6 Cancer Progression Research Center, National Yang Ming Chiao Tung University, Taipei 11221, Taiwan

**Keywords:** invasiveness, lung adenocarcinoma, *O*-GlcNAc transferase, *O*-GlcNAcylation, SAM68

## Abstract

**Simple Summary:**

Lung cancer claims the most lives annually among cancers; to date, invasion and metastasis still pose challenges to effective treatment. *O*-GlcNAcylation, an enzymatic modification of proteins after biosynthesis, modulates the functions of many proteins. Aberrant *O*-GlcNAcylation is linked to pathogenic mechanisms of cancer, including invasion and metastasis. However, little is known about the profile of *O*-GlcNAcylated proteins involved in cancer aggressiveness. Here, by comparing profiles of *O*-GlcNAcylated proteins from two lung cancer cell lines different in their invasive potential, we identified candidates for *O*-GlcNAcylated proteins that may be involved in cancer aggressiveness. One of these candidates, SAM68, was further characterized. Results confirmed *O*-GlcNAcylation of SAM68; functional analyses on SAM68 with mutations at *O*-GlcNAcylation sites suggested a role of *O*-GlcNAcylated SAM68 in modulating lung cancer cell migration/invasion. Future elucidation of the functional significance of differential *O*-GlcNAcylation of proteins identified in this study may provide new insights into mechanisms of lung cancer progression.

**Abstract:**

*O*-GlcNAcylation is a reversible and dynamic post-translational protein modification catalyzed by *O*-GlcNAc transferase (OGT). Despite the reported association of *O*-GlcNAcylation with cancer metastasis, the *O*-GlcNAc proteome profile for cancer aggressiveness remains largely uncharacterized. Here, we report our comparative *O*-GlcNAc proteome profiling of two differentially invasive lung adenocarcinoma cell lines, which identified 158 down-regulated and 106 up-regulated candidates in highly invasive cells. Among these differential proteins, a nuclear RNA-binding protein, SAM68 (SRC associated in mitosis of 68 kDa), was further investigated. Results showed that SAM68 is *O*-GlcNAcylated and may interact with OGT in the nucleus. Eleven *O*-GlcNAcylation sites were identified, and data from mutant analysis suggested that multiple serine residues in the *N*-terminal region are important for *O*-GlcNAcylation and the function of SAM68 in modulating cancer cell migration and invasion. Analysis of clinical specimens found that high SAM68 expression was associated with late cancer stages, and patients with high-OGT/high-SAM68 expression in their tumors had poorer overall survival compared to those with low-OGT/low-SAM68 expression. Our study revealed an invasiveness-associated *O*-GlcNAc proteome profile and connected *O*-GlcNAcylated SAM68 to lung cancer aggressiveness.

## 1. Introduction

Lung cancer is by far the most common cause of cancer death worldwide; globally reported deaths from lung cancer were around 180,000 in 2020 [1]. The two major diagnosed classes of lung cancers are non-small-cell lung cancer (NSCLC, ~85%) and small-cell lung cancer (~15%) [2]. Adenocarcinoma accounting for ~40% and ~70% of the male and female lung cancers in Taiwan, is the prevailing histological type of NSCLC and the most common primary lung malignancy in patients who have never smoked [2,3,4]. The prognosis of lung cancer remains poor, and the overall 5-year survival rate is only 10–20% [1]. The mortality mainly results from metastasis, which is a progression requiring cancer cells to undergo intravasation, survival in circulation, extravasation, and colonization [5]. Cancer invasion—a process involving disruption of the surrounding extracellular matrix and increasing cell motility—is the initial and a critical step for metastasis. However, a complete understanding of the molecular mechanisms underlying the regulation of cancer invasiveness remains elusive.

*O*-GlcNAcylation is the addition of a single β-*N*-acetylglucosamine (GlcNAc) to the hydroxyl group of serine/threonine residues in many nuclear and cytoplasmic proteins [6]. Cycling of this *O*-linked GlcNAc (*O*-GlcNAc) modification is regulated by a pair of evolutionary conserved enzymes: *O*-GlcNAc transferase (OGT) catalyzes the attachment of the GlcNAc moiety from UDP-GlcNAc to target proteins [7,8], whereas *O*-GlcNAcase (OGA) removes *O*-GlcNAc from *O*-GlcNAcylated proteins [9,10]. *O*-GlcNAcylation is an abundant and dynamic post-translational modification involved in many crucial cellular processes, including regulation of gene expression, signal transduction, metabolism, and cell cycles [11,12,13,14]. Ever-growing evidence indicates that dysregulation of cellular *O*-GlcNAc levels is associated with various diseases, including diabetes, neurodegenerative disorders, and cancer [13,14,15,16].

Aberrant *O*-GlcNAcylation has been linked to pathogenic mechanisms of cancer, including cell proliferation, survival, invasion, and metastasis [17]. Regulation of *O*-GlcNAc modification on specific proteins can modulate the migration and invasiveness of tumor cells. For instance, *O*-GlcNAcylation on Ser-112 of Snail, an important transcription factor involved in promoting the epithelial-mesenchymal transition and cancer cell invasiveness, can increase the stability of Snail protein and promote tumor invasion through repression of E-cadherin expression [18]. In breast cancer cells, loss of *O*-GlcNAcylation on Ser-108 of cofilin results in destabilization of invadopodia and impairs cancer cell invasion [19], and *O*-GlcNAcylation on Ser-408 of TAK1 binding protein 3 (TAB3) contributes to TAB3-mediated promotion of cancer cell migration and invasion by activating NF-ĸB [20]. Nevertheless, an overall profile of *O*-GlcNAcylated proteins that are involved in the pathogenesis of cancer, especially in invasion/metastasis, is still unclear.

We previously found that OGT expression is an independent prognostic factor in patients with lung adenocarcinoma [21]. It has been reported that knockdown of OGT expression in A549 lung cancer cells decreases their invasiveness in vitro [22]. TGF-β-induced epithelial-mesenchymal transition of A549 cells is accompanied by significantly increased OGT expression and cellular *O*-GlcNAcylation [23]. These observations suggest that *O*-GlcNAcylated proteins likely contribute to lung cancer invasion/metastasis. In light of this, this study aimed at uncovering *O*-GlcNAcylated proteins that modulate the invasiveness of lung cancer cells. We employed two lung adenocarcinoma cell lines with different invasive abilities; using label-free Mass spectrometry (MS) to analyze wheat germ agglutinin (WGA)-enriched proteins from these cells, we profiled the *O*-GlcNAc proteomes to identify *O*-GlcNAcylated proteins with differential abundance in the two cell lines. Among the identified proteins, we further investigated the SRC associated in mitosis of the 68-kDa (SAM68) protein to characterize its *O*-GlcNAcylation and its role in the migration/invasion of lung cancer cells.

## 2. Materials and Methods

### 2.1. Cell Lines and Cell Culture

Human lung adenocarcinoma cell lines CL1-1 and CL1-5 and A549 cells were maintained in RPMI-1640 (Gibco, Grand Island, NY, USA) supplemented with heat-inactivated 10% (*v*/*v*) fetal bovine serum (FBS) and 1% (*v*/*v*) penicillin/streptomycin. Human embryonic kidney 293T cells were maintained in Dulbecco’s modified Eagle’s medium (DMEM; Gibco, Grand Island, NY, USA) containing 10% (*v*/*v*) FBS. CL1-1 and CL1-5 cells were kindly provided by Prof. Pan-Chyr Yang from National Taiwan University, Taiwan. A549 and 293T cells, which were originally from Bioresource Collection and Research Center, Taiwan, were kindly gifted by Prof. Fung-Fang Wang from National Yang-Ming University, Taiwan. All cells were cultured at 37 °C in a humidified atmosphere with 5% CO_2_.

### 2.2. Nuclear/Cytosolic Fractionation

CL1-1 and CL1-5 cells were trypsinized to allow detachment from culture dishes, washed twice with PBS, and lysed in solution A (20 mM Tris, pH 7.5, 3 mM MgCl_2_, 1 mM EDTA, 0.1% Triton X-100, and protease inhibitors) at 1 × 10^7^ cells/mL on ice for 30 min. Lysates were centrifuged at 1500× *g* for 5 min, and supernatants were collected as the cytosolic fractions. Pellets were washed twice with solution A, suspended, vortexed in solution B (20 mM HEPES, pH 7.9, 25% Glycerol, 1% Triton X-100, and protease inhibitors), and incubated on ice for 30 min; after centrifugation at 15,000× *g* for 15 min, supernatants were collected as the nuclear fractions.

### 2.3. Enrichment of O-GlcNAcylated Proteins

For the enrichment of *O*-GlcNAcylated proteins, we used the Glycoprotein Isolation Kit (WGA #89805, Thermo Scientific, Waltham, MA, USA) following the manufacturer’s protocol. Briefly, 50% wheat germ agglutinin (WGA) resin slurry (200 μL/column) was applied to spin columns and washed twice with the Binding/Wash Buffer. Samples of nuclear or cytosolic fractions (1.5 mg/column) were added to the columns and incubated for 15 min at room temperature (RT) with end-over-end mixing using a rotator. WGA resin was collected by centrifugation and washed four times with the Binding/Wash Buffer. WGA-captured proteins were eluted twice by incubating the resin with 200 μL of elution buffer for 10 min and spinning at 1000× *g* for 1 min, and eluate from the same column was pooled and concentrated by using an Amicon Ultra-0.5, 3 KDa (Millipore, Billerica, MA, USA). WGA-captured glycoproteins were quantified using Bradford assays (Bio-Rad Laboratories, Richmond, CA, USA).

### 2.4. In-Gel Digestion and Liquid Chromatography (LC)-Mass Spectrometry (MS)/MS Analysis

Proteins eluted from WGA resin were separated by 10% SDS-PAGE and stained using the VisPRO 5 min Protein Stain Kit (Visual Protein Biotechnology Corporation, Taipei, Taiwan). Each sample lane was excised, cut into pieces, and dried in a SpeedVac (Thermo Electron, Waltham, MA, USA). Dried gel pieces of each sample were first incubated in 1% β-mercaptoethanol in 25 mM NH_4_HCO_3_ for 20 min at RT in the dark and further incubated for 20 min after an equal volume of 5% 4-vinylpyridine in 25 mM NH_4_HCO_3_ and 50% acetonitrile was added. After the removal of solvent, gel slices were washed in 25 mM NH_4_HCO_3_ for 10 min and dried in a SpeedVac. For in-gel digestion, gel pieces were incubated with cold 0.1% modified trypsin (Promega, Madison, WI, USA) first at 4 °C for 20 min and subsequently at 37 °C overnight. The digest solution containing tryptic peptides was collected, dried in a Speed-Vac, and stored at −20 °C until further analysis.

Each trypsin-digested sample was suspended in 20 µL of 0.1% formic acid and analyzed using the nanoACQUITY™ system (Waters, Milford, MA, USA), which was connected to an Orbitrap Elite hybrid mass spectrometer equipped with a nanoelectrospray ionization source (Thermo Scientific, Waltham, MA, USA). Peptides were separated by reverse-phase LC using a BEH-C18 column (25 cm × 75 µm, Waters, Milford, MA, USA) with a segmented gradient of 5% to 35% acetonitrile in 0.1% formic acid for 210 min at a flow rate of 300 nl/min. Eluted peptides were ionized at a spray voltage of 1.7 kV and introduced into the Orbitrap Elite for MS analysis in the positive ion mode with data-dependent acquisition (2.0 Da isolation width). A full MS scan was set at a resolution of 30,000 at *m*/*z* 400. Spectrum data of peptides were obtained by full-mass survey scan (*m*/*z* range: 350–1600). MS/MS scan by collision-induced dissociation was performed on 10 most intense multiply charged ions (2^+^ and 3^+^).

The mass spectrometry proteomics data have been deposited in the ProteomeXchange Consortium (http://proteomecentral.proteomexchange.org, accessed on 10 November 2021) via the PRIDE [24] partner repository with the dataset identifier PXD029627 and DOI 10.6019/PXD029627.

### 2.5. Protein Identification and Analysis

For protein identification, the acquired MS data were analyzed by using the Peaks7.5 Studio software for proteomics (Bioinformatics Solutions, Waterloo, Canada) to search against the UniProt human protein database (containing 192,901 sequences; released in January 2021; http://www.uniprot.org/, accessed on 15 January 2021). Search parameters were as follows: enzyme, trypsin; parent and fragment mass error tolerance, 50 ppm and 0.8 Da, respectively; allowing two missed cleavages, oxidation on methionine (+15.99 Da), and carbamidomethylation on cysteine (+57.02 Da) as variable modifications. The average local confidence (ALC) was >80%. A decoy database was constructed to estimate the false discovery rate (FDR), and FDR was controlled at <0.1%. A protein was identified when at least one unique peptide was matched. Protein quantitation based on the MS spectra was performed with in-house software [25]. *O*-GlcNAcylated peptide sequences and sites of WGA-captured proteins were identified using the PeaksPTM module of the PEAKS 7.5 software (Bioinformatics Solutions, Waterloo, Canada). Possible ions (e.g., b, y, y-NH_3_, or b-H_2_O) of modified fragment peptides in the MS spectra were manually labeled. Differential WGA-bound nuclear and cytosolic glycoproteins were analyzed using the PANTHER classification system (http://www.pantherdb.org/, accessed on 13 February 2019) [26] for molecular function, biological process, and protein class-based gene ontology. Pathway analysis was performed using Ingenuity Pathway Analysis (IPA; Ingenuity Systems, Redwood City, CA, USA) based on experimental observations of the target genes reported in the literature to determine differentially regulated signaling networks with significance calculated using Fisher’s exact test (*p*-value < 0.05).

### 2.6. Western Analysis

Samples of nuclear and cytosolic lysates and WGA flow-through, wash, and elution fractions were resolved in 10% SDS-PAGE gels and transferred onto polyvinylidene fluoride transfer membranes (PVDF, Pall Corporation, East Hills, NY, USA). Membranes were blocked in TBS-T buffer containing 5% BSA for 1 h at RT and were then incubated with primary antibodies overnight at 4 °C. Antibodies used include: anti-*O*-GlcNAc CTD110.6 (1:2000; MMS-248R; BioLegend, CA, USA), anti-IQGAP1 (1:1000; sc-10792; Santa Cruz Biotechnology Inc., Santa Cruz, CA, USA), anti-LSD1 (1:1000; C69G12; Cell Signaling Technology, Beverly, MA, USA), anti-MTA2 (1:1000; ab50209; Abcam, Cambridge, MA, USA), anti-SAM68 (1:5000; sc-333; Santa Cruz Biotechnology, Inc.), anti-B23 (1:1000; sc-6013-R; Santa Cruz Biotechnology Inc.), anti-Rock1 (1:1000; sc-17794; Santa Cruz Biotechnology Inc.), anti-OGT (1:2000; 11576-2-AP; Proteintech, Rosemont, IL, USA), anti-PARP1 (1:1000; GTX100573; GeneTex, Hsinchu City, Taiwan), anti-β-Actin (1:20000; NB600-501; Novus Biologicals, Centennial, CO. USA), anti-α-Tubulin (1:1000; T9026; Sigma-Aldrich, St. Louis, MO, USA), and anti-Flag (1:1000; F3165; Sigma-Aldrich). Rabbit anti-α-Enolase antibody was kindly provided by Prof. Neng-Yao Shih from the National Health Research Institutes, Taiwan. After washing, membranes were incubated with appropriate horseradish peroxidase HRP-conjugated secondary antibodies for 1 h at RT, and protein signals were visualized using a chemiluminescence ECL kit (HyCell International Co., Ltd., Taipei, Taiwan).

### 2.7. Co-Immunoprecipitation

CL1-5 cells were washed in PBS twice and treated with or without 1 mM crosslinker dithiobis (succinimidyl propionate) (DSP; Thermo Scientific #22585) for 15 min at RT. The reaction was quenched in 20 mM Tris-base pH 7.5 for 10 min at RT. Cells were harvested and suspended in the lysis buffer (150 mM NaCl, 0.5% Sodium deoxycholate, 1% NP-40, 0.1% SDS) with a protease inhibitor cocktail, gently passed through a 26-gauge needle 20 times, vortexed for 90 s, and then incubated on ice for 30 min. After centrifugation at 12,500 rpm in a microfuge for 10 min at 4 °C, the supernatant was collected and the protein concentration was measured by Bradford assays (Bio-Rad Laboratories, Richmond, CA, USA). Then, 20 μL of protein G magnetic beads was incubated with 1 mg of lysates and l μg of anti-OGT, anti-SAM68 or control IgG antibodies at 4 °C overnight. The beads were washed with lysis buffer three times and boiled in β-mercaptoethanol-containing sample buffer at 100 °C for 10 min, and proteins were subjected to SDS-PAGE and Western analysis.

### 2.8. Immunofluorescence Staining

CL1-5 cells were transiently transfected with a plasmid expressing Flag-tagged SAM68 or Myc-tagged OGT. Cells cultured on coverslips were fixed with 4% paraformaldehyde in PBS for 10 min, permeabilized with 0.5% Triton X-100 in PBS for 5 min, and blocked with 10% FBS in PBS for 30 min at RT. After PBS washing, fixed cells were incubated with primary antibodies, including anti-OGT (1:200; 11576-2-AP; ProteinTech), anti-SAM68 (1:200; sc-333; Santa Cruz Biotechnology Inc.), anti-Myc (1:100; MMS-150P; Covance, Richmond, CA, USA) or anti-Flag (20 μg/mL; F3165; Sigma-Aldrich), at 4 °C overnight. Cells were washed again in PBS and incubated with DAPI and appropriate rhodamine- or FITC-conjugated secondary antibodies at RT for 1 h. Finally, the slides were washed in PBS and mounted in mounting solution. Cells were examined under a confocal fluorescence microscope.

### 2.9. Knockdown of SAM68-Encoding KHDRBS1 by Lentivirus-Delivered shRNAs

Plasmids for the expression of shRNAs targeting *KHDRBS1* (which encodes SAM68) (TRCN0000000044, designated as #1, and TRCN0000000048, designated as #2) were obtained from the National RNAi Core Facility Platform located at the Institute of Molecular Biology/Genomic Research Center, Academia Sinica, Taipei, Taiwan. Virus packaging and target cell transduction were performed as described previously [27].

### 2.10. Transwell Migration and Invasion Assays

Cell culture inserts with 8-μm pores (Millicell^®^; Merck Millipore Ltd., Billerica, MA, USA) were used. For migration assay, 2 × 10^4^ cells/well in 1% FBS-containing medium were placed in the upper chambers; the lower chambers were filled with 10% FBS-containing medium, and the assay was performed at 37 °C for 6 h. For invasion assay, the cell culture inserts were coated with matrigel (40 μg/well), and 2 × 10^4^ cells/well in 10% FBS-containing medium were seeded on top of each filter insert; medium supplemented with 10% serum was added to the lower chambers, and the assay was performed at 37 °C for 24 h. At the end of the assays, the inserts were fixed in 100% methanol for 20 min, air dried, and stained with Giemsa Stain (Sigma-Aldrich) overnight. The inner side of the inserts was wiped with cotton swaps, and the cells that migrated or invaded through the insert were counted under a light microscope.

### 2.11. Immunohistochemical (IHC) Staining

A tissue microarray (TMA) that contained 174 patients with lung adenocarcinoma at various stages was analyzed; these patients underwent tumor resection at Taipei Veterans General Hospital (Taipei-VGH) between 2002 and 2006. The collection and usage of clinical samples complied with the regulations of the Taipei-VGH Institutional Review Board (IRB No. 2021-04-011BCF). The stage of lung adenocarcinoma was determined according to the Union for International Cancer Control/American Joint Committee on Cancer TNM classification. IHC staining was performed as described previously [21]. Briefly, after rehydration, antigen retrieval, and peroxidase blocking, TMA sections were incubated with anti-SAM68 (1:50; LifeSpan BioSciences, Inc., Seattle, WA, USA), anti-OGT (1:50; ProteinTech, Chicago, IL, USA) or anti-*O*-GlcNAc (1:200; Thermo Fisher Scientific, Waltham, MA, USA) antibodies overnight at 4 °C and subsequently, after washing in phosphate-buffer saline (PBS), with peroxidase-labeled secondary antibody for 1 h at RT. Sections were then incubated with diaminobenzidine, washed, and counterstained with hematoxylin before being mounted. The staining was examined by pathologists and semi-quantitatively scored as follows: 0 (no staining), 1 (weakly positive), 2 (moderately positive), and 3 (strongly positive); percentage scores were 0–100%. The IHC total score for each specimen was the intensity score multiplied by the percentage score. The median of the total scores was used as the cut-off value to categorize patients into high- and low-expression groups.

### 2.12. Statistical Analysis

Presented quantitative results are the mean ± standard deviation from at least three independent experiments. Comparisons were performed by two-tailed unpaired Student’s *t*-test. A *p* value < 0.05 was considered statistically significant.

## 3. Results

### 3.1. Differential O-GlcNAcylated Proteins in Lung Adenocarcinoma Cell Lines with Low and High Invasiveness

**Figure 1:**
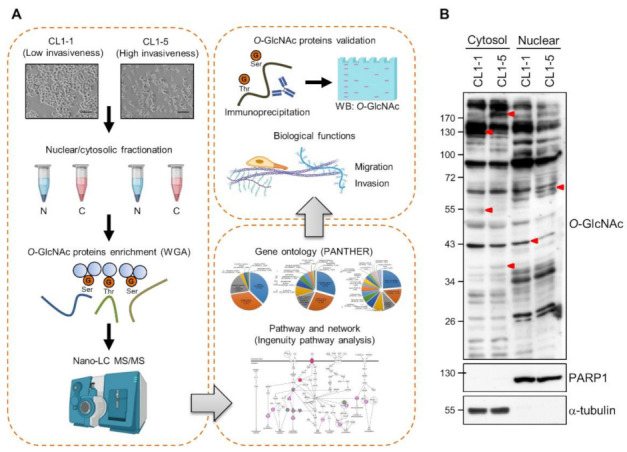
Profiling of the *O*-GlcNAc proteomes of two differentially invasive lung adenocarcinoma cell lines. (**A**) A schematic workflow of this study. Lysates were prepared from subconfluent lowly invasive CL1-1 and highly invasive CL1-5 cells and fractionated into nuclear (N) and cytosolic (**C**) extracts. *O*-GlcNAcylated proteins were enriched using resin-bound wheat germ agglutinin (WGA). Tryptic peptides of WGA-bound proteins were subjected to nano-LC-MS/MS. Reproducibly identified differential WGA-bound proteins were further analyzed in silico using bioinformatics tools including PANTHER Gene Ontology and Ingenuity pathway analysis. Selected differential WGA-bound proteins were further validated and characterized for roles in cancer cell migration and invasion. (**B**) Representative results of Western analysis of cytosolic and nuclear exacts of CL1-1 and CL1-5 cells. Proteins were separated by 10% SDS-PAGE and subjected to Western blotting using antibodies for *O*-GlcNAc, PARP1 (a nuclear protein marker) and α-tubulin (a cytosolic protein marker). Arrowheads point to examples of differential bands between CL1-1 and CL1-5 samples. Detailed information about the Western blotting can be found in Figure S7. PANTHER, Protein ANalysis THrough Evolutionary Relationships; MS, Mass Spectrometry; orange circled G, *O*-GlcNAc.

Aiming at identifying *O*-GlcNAcylated proteins related to cancer invasiveness, we employed CL1-1 and CL1-5, which are two well-established human lung adenocarcinoma cell lines with differential invasiveness (Figure 1A). These cell lines have been established from one human lung adenocarcinoma clone through sequential in vitro Transwell enrichment for invasive subpopulations; CL1-1 was established after one round while CL1-5 after five rounds of selection [28]. CL1-1 cells exhibit a typical epithelial-type morphology and tend to form cell clusters, while CL1-5 cells display a spindle-shaped fibroblast-like morphology with less cohesiveness; accordingly, CL1-1 cells have low invasiveness while CL1-5 cells are highly invasive. The isogenic nature of these two cell lines offers an advantage in this comparative study for invasiveness-associated proteomes, as proteomic differences caused by distinct genetic backgrounds would be minimized. When cytosolic and nuclear fractions of lysates from CL1-1 and CL1-5 cells were examined for *O*-GlcNAcylated proteins by Western analysis using *O*-GlcNAc-recognizing antibodies, some distinct differential *O*-GlcNAcylated protein bands were noted (Figure 1B), indicating that cells with different degrees of invasiveness may have differential *O*-GlcNAc proteomes.

### 3.2. Identification and Validation of Differential WGA-Bound Glycoproteins in Lung Adenocarcinoma Cell Lines with Low and High Invasiveness

**Figure 2:**
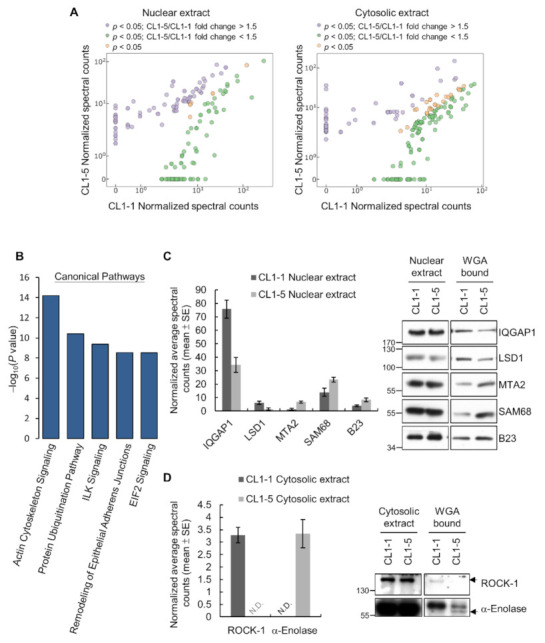
Differential WGA-bound glycoproteins identified in CL1-1 and CL1-5 cells. (**A**) Scatter plots of the normalized spectral counts of proteins with statistically significant (Student’s *t*-test, *p*-value < 0.05) fold changes in their amounts in WGA-enriched nuclear or cytosolic fractions from CL1 and CL1-5 cells. (**B**) Top canonical pathways identified by IPA of differential WGA-bound glycoproteins. (**C**–**D**) Validation of representative differential WGA-bound nuclear (**C**) and cytosolic (**D**) glycoproteins by Western analysis. Shown on the left are bar graphs of average normalized spectral counts listed in Tables S1 (**C**) and S2 (**D**); N.D., not determined. Representative Western results are shown on the right. Detailed information about the Western blotting can be found in Figure S8.

Towards identifying *O*-GlcNAcylated proteins with differential amounts in two cell lines, we employed WGA to perform enrichment of *O*-GlcNAcylated proteins. Western analysis showed that *O*-GlcNAcylated proteins from nuclear/cytosolic fractions were indeed enriched in the WGA elution fractions (Figures S1A,B and S14). We next performed in-gel trypsin digestion on samples of WGA elution fractions, and the resulting tryptic peptide mixtures from CL1-1 and CL1-5 samples were independently analyzed in triplicate by nano-LC-MS/MS for protein identification. Comparing the mass spectrometry data and using a threshold of >1.5-fold change between the two cell lines, we reproducibly identified 63 down-regulated and 62 up-regulated candidates for nuclear *O*-GlcNAcylated proteins, and 95 down-regulated and 44 up-regulated cytosolic ones in CL1-5 cells (Student’s *t*-test, *p*-value < 0.05) (Figure 2A and Tables S1 and S2).

We further performed gene ontology (GO) analysis and Ingenuity Pathway Analysis (IPA) to identify functional networks and canonical pathways of these differential WGA-bound nuclear and cytosolic proteins. Results of GO slim analysis showed that the top identified protein classes were nucleic acid binding proteins, followed by cytoskeletal proteins, hydrolases, and enzyme modulators (Figure S2A); the top associated molecular functions were binding and catalytic activities (Figure S2B), and these proteins were mainly involved in the biological functions of metabolism, cellular process, and cellular component organization or biogenesis (Figure S2C). Results of IPA revealed canonical pathways and network functions that were significantly associated with the differential WGA-bound proteins (Figure 2B and Table S3). Interestingly, the top associated canonical pathway was “Actin Cytoskeleton Signaling”, which plays a pivotal role in cell migration (Table S4 and Figure S3); this finding is consistent with the notion that some differential *O*-GlcNAcylated proteins may be involved in promoting lung cancer cell migration. Moreover, the molecular and cellular functions of these glycoproteins identified by IPA were mostly associated with cellular growth, proliferation, survival, assembly, and organization (Table S5), which generally agreed with the findings of GO analysis.

Among the LC-MS/MS-identified candidates of differential *O*-GlcNAcylated proteins, we picked several that have been implicated in cancer cell migration, invasion or metastasis in the literature for validation by Western analysis. We examined the amounts of WGA-bound IQ Motif Containing GTPase Activating Protein 1 (IQGAP1), Lysine-specific histone demethylase 1 (LSD1), Metastasis Associated 1 Family, Member 2 (MTA2), Src-Associated in Mitosis 68 KDa Protein (SAM68), and Nucleophosmin (NPM) in nuclear fractions and the amounts of WGA-bound Rho-associated protein kinase 1 (ROCK-1) and α-Enolase in cytosolic fractions from CL1-1 and CL1-5 cells. For all the seven examined candidates, the Western results were consistent with the observed differences in their average normalized spectral counts in LC-MS/MS profiling (Figure 2C,D). To our knowledge, there are no reports describing the investigation of *O*-GlcNAc modification of any of these seven candidates. However, we found all seven of them listed as *O*-GlcNAcylated proteins in the *O*-GlcNAc Database v1.2 (https://www.oglcnac.mcw.edu, accessed on 14 Dec 2021), which is a human *O*-GlcNAcome database created by the Olivier-Van Stichelen lab at the Medical College of Wisconsin Department of Biochemistry using published *O*-GlcNAcome data [29]. In fact, out of the 264 differential WGA-bound proteins we identified, 246 (246/264; 93%) were listed as *O*-GlcNAcylated proteins in the *O*-GlcNAc Database v1.2 (Tables S1 and S2). Together, these results suggest that the profiling workflow was effective in identifying differential *O*-GlcNAcylated proteins in cancer cells with different degrees of invasiveness.

### 3.3. SAM68 Is an O-GlcNAcylated Protein Associated with OGT in Lung Cancer Cells

**Figure 3:**
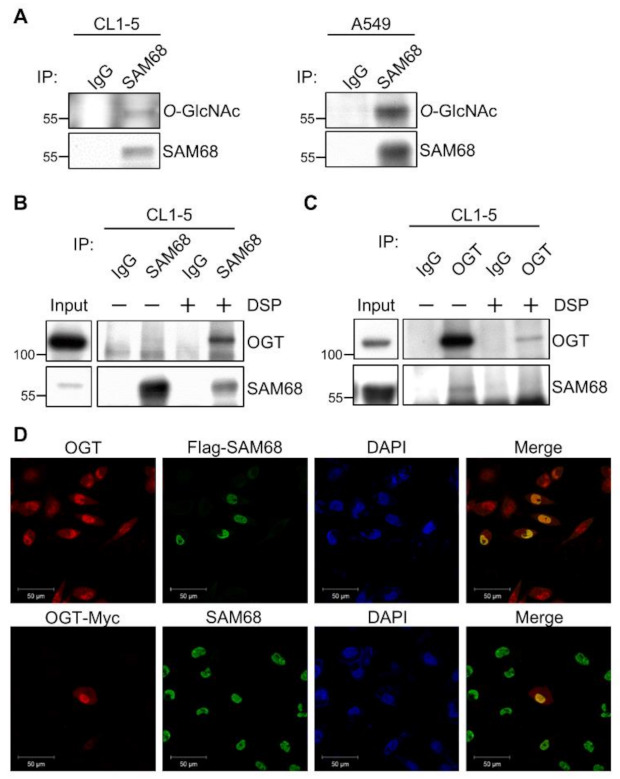
*O*-GlcNAcylation of SAM68 and its interaction with OGT. (**A**) *O*-GlcNAc Western analysis of immunoprecipitated SAM68. Total lysates from CL1-5 and A549 cells were subjected to immunoprecipitation with anti-SAM68 or IgG antibodies and subsequent Western blotting using the antibodies indicated. (**B**,**C**) Co-immunoprecipitation. Lysates from CL1-5 cells treated with or without the cross-linker DSP were subjected to immunoprecipitation using anti-SAM68 (**B**) or anti-OGT (**C**) antibodies, and immunoprecipitated proteins were analyzed by Western blotting with indicated antibodies. (**D**) Immunofluorescence cell staining. CL1-5 cells transiently expressing Flag-tagged SAM68 or Myc-tagged OGT were fixed and subjected to immune-staining using primary antibodies recognizing OGT, Flag, Myc, and SAM68 as indicated; DAPI was used to stain cell nuclei. Micrographs were captured under a fluorescence confocal microscope. Detailed information about the Western blotting can be found at Figure S9. OGT, *O*-GlcNAc transferase; DSP, dithiobis (succinimidyl propionate).

We chose SAM68 for further characterization of its *O*-GlcNAcylation and involvement in lung cancer aggressiveness. SAM68 is a nuclear RNA-binding protein belonging to the signal transduction and activation of RNA metabolism (STAR) family [30,31,32], and evidence in the literature has linked SAM68 to tumorigenesis and progression of different cancers [33,34,35,36,37]. Although SAM68 has been discovered as an *O*-GlcNAcylated protein in a high-throughput study [38], its *O*-GlcNAcylation remains uncharacterized. Given that a higher amount of SAM68 was pulled-down by WGA from the nuclear extract of CL1-5 cells than from that of CL1-1 cells, we sought to confirm the *O*-GlcNAc modification on SAM68 in lung cancer cells by immunoprecipitation of SAM68 from total cell lysates of CL1-5 and A549 cells and Western analysis using anti-*O*-GlcNAc antibodies. Results demonstrated that endogenous SAM68 was *O*-GlcNAcylated in both cell lines (Figure 3A). We explored the physical interaction between SAM68 and OGT by co-immunoprecipitation. In the samples immunoprecipitated by anti-SAM68 antibodies but not in those by IgG, a faint signal of OGT was detected; considering that the interaction might be transient and/or weak, we employed the cross-linker dithiobis [succinimidylpropionate] (DSP), and the results showed that OGT was co-immunoprecipitated by SAM68-specific antibodies from lysates of DSP-treated CL1-5 cells (Figure 3B). Reciprocal experiments using anti-OGT antibodies for immunoprecipitation detected co-immunoprecipitated SAM68 in the sample of untreated cells but not in the sample of DSP-treated cells, probably because the antibodies immunoprecipitated much less OGT in the DSP-treated sample (Figure 3C). Furthermore, we conducted immunofluorescence staining to examine the localization of SAM68 and OGT in CL1-5 cells transiently expressing Flag-tagged SAM68 or Myc-tagged OGT. The signals of SAM68 and OGT were predominantly co-localized in the nucleus (Figure 3D). Collectively, these results confirmed that SAM68 is *O*-GlcNAcylated and suggested that it interacts with OGT mainly in the nucleus.

### 3.4. The N-Terminal Region of SAM68 Is Crucial for Its O-GlcNAcylation

**Figure 4:**
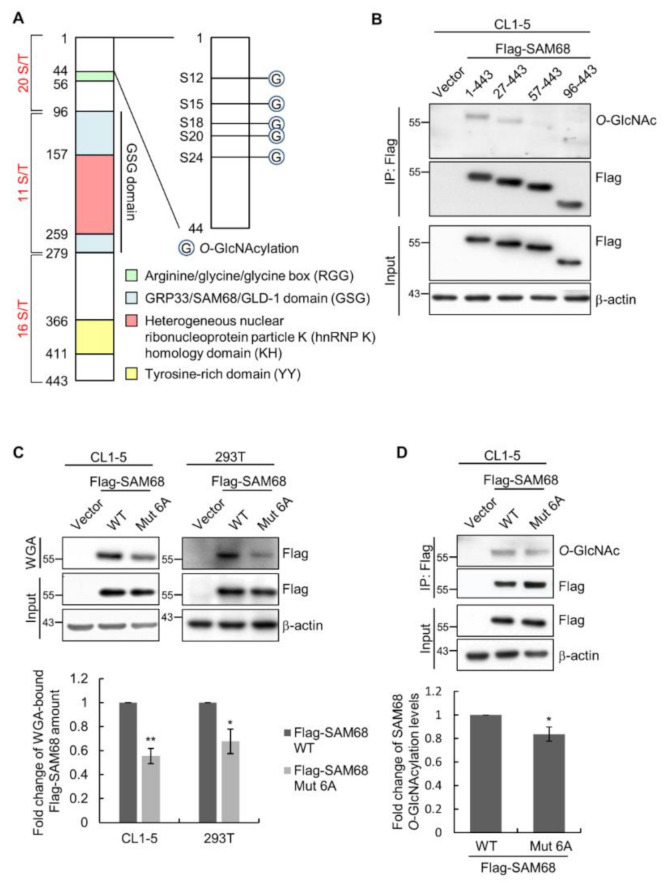
Characterization of SAM68 *O*-GlcNAcylation by truncation analysis and site-directed mutagenesis. (**A**) A schematic of the SAM68 domain structure. Numbers are aa positions. Total numbers of Ser/Thr (S/T) residues in indicated regions are shown. (**B**) Comparison of *O*-GlcNAcylation levels of full-length SAM68 (WT; 1-443 aa) and truncation mutants (indicated by remaining aa). Whole cell lysates from CL1-5 cells transfected with the control vector or SAM68 expression constructs were subjected to immunoprecipitation and Western analysis as indicated. (**C**,**D**) Comparison of *O*-GlcNAcylation levels of wild-type (WT) and a mutant SAM68 (Mut 6A; with S12A/S14A/S15A/S18A/S20A/S24A substitutions). CL1-5 and 293T cells were transfected for 24 hrs to express WT or Mut 6A before analysis. Representative Western results and quantitative data from multiple independent experiments are shown. (**C**) Lysates were subjected to WGA-pulldown and subsequent Western analysis. (**D**) Lysates were subjected to immunoprecipitation and subsequent Western analysis. All quantitative data shown are the means ± SD; *, *p* < 0.05; **, *p* < 0.01. Detailed information about the Western blotting can be found in Figures S10–S12.

SAM68 contains a total of 47 Ser/Thr residues in its sequence and most of them are located within the *N*-terminal 100 aa of the protein (Figure 4A). To explore the region important for *O*-GlcNAcylation in SAM68, we generated a series of *N*-terminal truncation mutants of SAM68; results of immunoprecipitation and Western analysis of these mutants showed that deletion of aa 1–26 significantly decreased the *O*-GlcNAcylation of SAM68 and deletion of aa 1–56 or aa 1–95 further diminished the modification (Figure 4B), indicating the importance of the *N*-terminal region for SAM68 *O*-GlcNAcylation. We next surveyed the *O*-GlcNAc-modified residues in SAM68 by MS/MS analysis of immunoprecipitated SAM68 or WGA-bound proteins, and results from multiple experiments revealed 11 *O*-GlcNAcylation sites (Figure S4). Notably, in the *N*-terminal region mapped to be important for SAM68 *O*-GlcNAcylation, S12, S15, S18, S20, and S24 were confirmed to be *O*-GlcNAcylation sites. We further investigated the aa 1–26 region by preparing single-site mutants with individual Ser residues in this region replaced with Ala; results showed that all generated single-site mutants still retained significant amounts of *O*-GlcNAcylation on SAM68 (Figures S5 and S15). We then prepared a multiple-site mutant 6A (S12A/S14A/S15A/S18A/S20A/S24A). When lysates from CL1-5 and 293T cells transfected with plasmids expressing wild-type (WT) or 6A mutant SAM68 were subjected to WGA pull-down and Western analysis, we found the amount of WGA-bound mutant was significantly smaller than that of WGA-bound WT SAM68 in both cell lines (Figure 4C). Additionally, SAM68 immunoprecipitation combined with *O*-GlcNAc Western analysis of lysates from WT or 6A SAM68-expressing CL1-5 cells confirmed that *O*-GlcNAcylation on 6A mutant SAM68 was significantly lower than that on WT SAM68 (Figure 4D). These data together suggest that multiple serine residues in the *N*-terminal 26 aa region are involved in *O*-GlcNAcylation of SAM68.

### 3.5. O-GlcNAcylation Sites in the N-Terminal Region of SAM68 Are Important for Regulating Lung Cancer Cell Migration and Invasion

**Figure 5:**
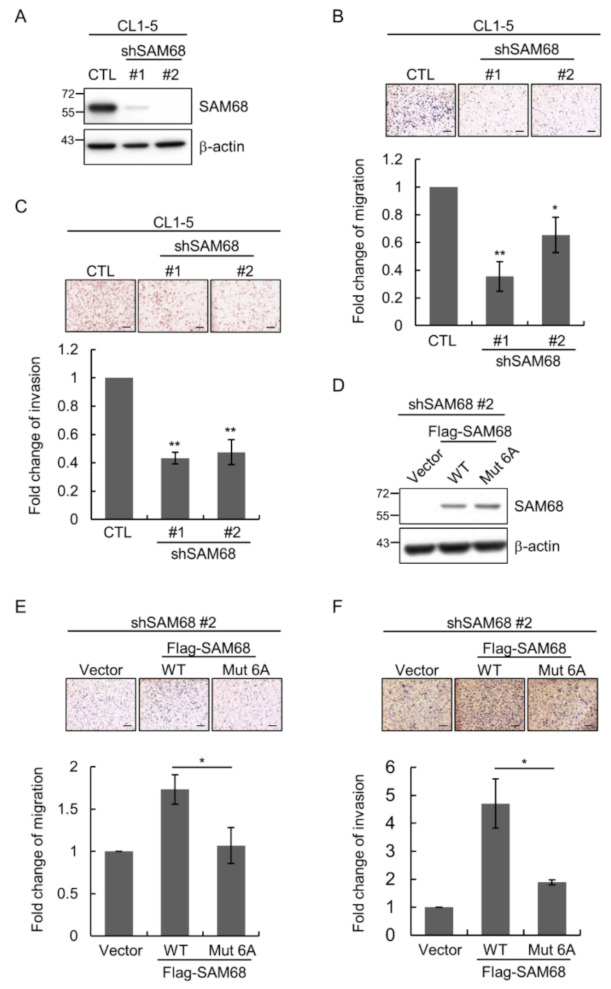
*O*-GlcNAcylation in the *N*-terminal region of SAM68 and its functional significance. (**A**–**C**) Two different *KHDRBS1*-targeting shRNAs (shSAM68 #1 and #2) were used to generate independent SAM68-knockdown CL1-5 clones. The LKO vector was used as an infection control (CTL). Cells were subjected to Western analysis (**A**), Transwell migration assays (**B**), and Transwell invasion assay (**C**). (**D**–**F**) CL1-5 shSAM68 #2 cells were transfected by the control vector or a construct to express Flag-tagged SAM68 WT or mutant 6A, and resulting cells were subjected to Western analysis (**D**), Transwell migration assays (**E**), and Transwell invasion assays (**F**). All quantitative data shown are the means ± SD of multiple independent experiments; *, *p* < 0.05; **, *p* < 0.01. Scale bar, 100 μm. Detailed information about the Western blotting can be found in Figure S13.

For investigating the role of SAM68 in lung cancer cell aggressiveness, we obtained CL1-5 clones in which the expression of SAM68 was stably suppressed by two different SAM68-targeting shRNAs (shSAM68 #1 and shSAM68 #2) (Figure 5A). Compared to the control cells, silencing of SAM68 expression markedly decreased the migration (Figure 5B) and invasion (Figure 5C) of CL1-5 cells in Transwell assays. To explore the importance of *O*-GlcNAc modifications in the *N*-terminal region of SAM68 for regulating cancer cell migration and invasion, we expressed the WT and mutant 6A of SAM68 in shSAM68 #2 cells (Figure 5D). Transwell migration assays demonstrated that shSAM68 #2 cells with reconstituted expression of WT but not mutant SAM68 showed increased migration compared to the vector control cells (Figure 5E). Similarly, reconstitution of SAM68 expression in shSAM68 #2 cells increased the invasive capability of cells, but this effect was significantly blunted if SAM68 mutant 6A was used for reconstitution (Figure 5F). Together, these findings are consistent with the notion that *O*-GlcNAcylation in the *N*-terminal region of SAM68 may promote lung cancer aggressiveness by enhancing the migratory and invasive abilities of cancer cells.

### 3.6. Association of SAM68 Expression with Cancer Stage and Clinical Outcome of Patients with Lung Adenocarcinoma

**Figure 6:**
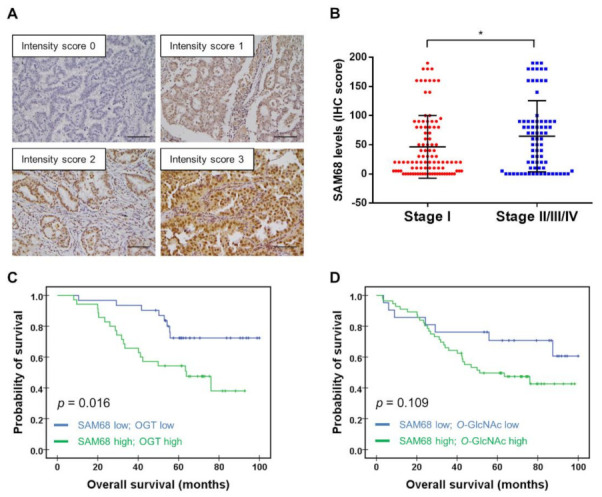
Association of SAM68 expression with lung cancer stage and patient outcome. (**A**) Analysis of SAM68 protein expression in clinical specimens of lung adenocarcinoma by immunohistochemistry (IHC). Shown are images representing different IHC staining intensity scores: negative (0), weak (1), moderate (2), and strong (3). Scale bar, 100 μm. (**B**) Analysis of SAM68 expression in lung adenocarcinoma tissues in relation to cancer stage. (**C**–**D**) Kaplan-Meier survival analysis of patients with lung adenocarcinoma categorized according to SAM68/OGT (**C**) or SAM68/*O*-GlcNAc (**D**) levels. *p*-values were derived from the log-rank test; *, *p* < 0.05.

Clinical significance of SAM68 in lung cancer was previously suggested by studies showing upregulation of *KHDRBS1* transcript (which encodes SAM68) or SAM68 protein levels in NSCLC or lung adenocarcinoma cancerous tissues [37,39,40,41]; however, association with poor outcome of patients with adenocarcinoma was only shown for high *KHDRBS1* transcript levels. To explore the prognostic association at the protein level, we examined a cohort of 174 patients with lung adenocarcinoma by performing immunohistochemistry (IHC) analysis for SAM68 expression on tissue microarrays constructed using specimens from these patients. The IHC result was scored by pathologists based on the intensity (Figure 6A) and percentage of staining. When the IHC results were analyzed in relation to clinical data, we found that tissues of lung adenocarcinoma at late stages (stage II–IV) exhibited higher levels of SAM68 than those at stage I (Figure 6B and Table S6). However, when we categorized patients into low- and high-SAM68 expression subgroups based on the median IHC score and performed Kaplan-Meier analysis, the difference in the overall survival of these two subgroups did not reach statistical significance (Figure S6). Considering our above-mentioned results suggesting an important role of SAM68 *O*-GlcNAcylation in modulating cancer cell migration and invasion, we further categorized the patients according to OGT and *O*-GlcNAc IHC scores and compared the outcome between subgroups. Kaplan-Meier analysis revealed that the high-SAM68/high-OGT subgroup had shorter overall survival than the low-SAM68/low-OGT subgroup (Figure 6C, *p* = 0.016, log-rank analysis), but no significant difference was observed between the high-SAM68/high-*O*-GlcNAc and low-SAM68/low-*O*-GlcNAc subgroups (Figure 6D, *p* = 0.109).

## 4. Discussion

Evidence indicates that *O*-GlcNAcylation plays important roles in tumor proliferation, resistance to apoptosis, metabolism, and metastasis; many oncogenic factors or tumor suppressors have been shown to be *O*-GlcNAcylated proteins [13,42,43]. Hence, identification of cancer-associated *O*-GlcNAcylated proteins should aid in our understanding of molecular mechanisms underlying cancer pathogenesis. To date, only a handful of publications describe proteomic profiling of cancer-related *O*-GlcNAcylated proteins associated with breast cancer [44,45], colorectal cancer [46], and cholangiocarcinoma [47]. To our knowledge, invasion-associated *O*-GlcNAc proteomes of human lung cancer cells have not been explored. In this study, we used WGA enrichment and mass spectrometry to reveal *O*-GlcNAcylated proteins that may relate to lung adenocarcinoma aggressiveness. Among the identified candidate *O*-GlcNAcylated proteins, SAM68 was validated to be *O*-GlcNAcylated and was shown to be associated with OGT in the nucleus of lung cancer cells. Additionally, we obtained evidence suggesting that *O*-GlcNAcylated SAM68 promotes the migratory and invasive abilities of lung cancer cells. Furthermore, IHC analysis of clinical specimens revealed the association of simultaneous high expression of SAM68 and OGT with poor patient outcome.

Although we were able to identify differential WGA-bound proteins, the global *O*-GlcNAcylation level of nuclear/cytosolic proteins appeared to be generally similar between the two differentially invasive lung adenocarcinoma cell lines in Western analysis. In a study that identified *O*-GlcNAcylated proteins in invasive ductal breast carcinomas with or without lymph node metastasis [45], it was also noted that the *O*-GlcNAcylation status of individual proteins was independent of the overall *O*-GlcNAcylation levels in metastatic and non-metastatic invasive ductal breast carcinomas. Moreover, our previous IHC study found no association between the global *O*-GlcNAcylation status and the outcome of patients in lung adenocarcinoma [21]. These findings together highlight the importance of exploring changes in *O*-GlcNAc modification on specific proteins in understanding the molecular mechanisms of cancer progression.

Our profiling identified 158 down-regulated and 106 up-regulated putative *O*-GlcNAcylated proteins in the highly invasive compared to the lowly invasive lung adenocarcinoma cells. Results of Gene ontology (GO) analysis of these proteins generally reflect the previously understood functions of *O*-GlcNAcylated proteins. For example, the largest protein class of identified candidates in this study was “nucleic acid binding” (Figure S2C), which is well in line with the fact that OGT can localize to the nucleus and that numerous *O*-GlcNAcylated proteins are involved in transcriptional regulation [14,48]. GO Biological Process analysis identified “metabolic process” as the major process associated with the identified differential proteins (Figure S2B), which is consistent with the well-established role of cellular *O*-GlcNAcylation in metabolic regulation; as cellular *O*-GlcNAcylation status is influenced by nutrient availability via the hexosamine biosynthetic pathway (HBP), *O*-GlcNAcylation is considered a nutritional sensor and metabolic regulator [12]. Many studies have reported the role of *O*-GlcNAc modification in regulating cancer metabolism by modifying signaling proteins, metabolic enzymes, and transcription factors [17]. Our finding of the metabolic process as a major function associated with the invasiveness-related *O*-GlcNAc proteome emphasizes the scenario in which aberrant cancer metabolism affects tumor cell migration and invasion. It is established that metabolic reprogramming in cancer cells may lead to HIF-1α activation, which reduces E-cadherin expression and promotes the epithelial-mesenchymal transition and cancer cell invasion [49]. However, we did not identify HIF-1α in this study; the molecular basis linking *O*-GlcNAcylated protein-mediated modulation of cancer metabolism and lung cancer cell invasiveness requires further investigation. Importantly, the most significant canonical pathways associated with the invasiveness-associated *O*-GlcNAc proteome revealed in this study, which included actin cytoskeleton signaling, ILK signaling, and remodeling of epithelial adherens junctions as revealed by the IPA (Table S4), stress the role of *O*-GlcNAcylation in modulating the functions of key molecular players in these pathways. Future exploration of these *O*-GlcNAcylated proteins may expand our understanding of how *O*-GlcNAcylation regulates lung cancer cell invasion.

Our data here clearly demonstrated that the nuclear RNA-binding protein SAM68 is highly *O*-GlcNAcylated, with *O*-GlcNAc modification on multiple sites. SAM68 contains several defined domains in its protein structure [33]. The *N*-terminal region contains several Pro-rich motifs that may mediate the interaction with SH3 domain-containing proteins [50] and an RGG (Arginine-Glycine-Glycine) box [33]. The RNA-binding function of SAM68 is provided by an hnRNP-K homology domain (KH domain); additionally, there is a Tyr-rich (YY) domain near the C-terminus [33]. Among the 11 *O*-GlcNAcylated sites detected by MS analysis in our study, five (S12, S15, S18, S20, S24) locate in the N-terminal region but not in the Pro-rich motifs or the RGG box, two (T183, S202) are in the KH domain, three (T317, T324, T330) reside in the YY domain, and one (S422) is in the C-terminal region (Figure S4A). We noted that S18, S20, and T183 have also been identified as phosphorylated sites in a high-throughput study [51]. It will be of interest to investigate how these different types of protein modification of SAM68 functionally interact.

We focused our further investigation on the *N*-terminal region of SAM68 because analysis of truncation mutants suggested its importance for *O*-GlcNAcylation and because its role in SAM68 functions is not clear. The *N*-terminal region of SAM68 has a very high tendency of a disordered structure, as predicted by IUPred [50]. Intrinsically disordered regions are usually susceptible to diverse post-translational modifications and are characterized by marked conformational flexibility and structural plasticity to regulate protein-protein interactions [52,53]. Consistent with this notion, we identified multiple *O*-GlcNAcylated sites within this region of SAM68; future studies are obviously needed to elucidate the effect of *O*-GlcNAcylation of these sites on the interaction of SAM68 with its binding partners. Our finding that mutating *O*-GlcNAcylated sites in this region disrupt the function of SAM68 in the regulation of lung adenocarcinoma cell migration/invasion advocates a functional role of *O*-GlcNAcylation. However, as mentioned above that several identified *O*-GlcNAcylated Ser residues may also be phosphorylation sites, at this point we cannot rule out the possibility that phosphorylation may also modulate the function of SAM68 in regulating cancer cell aggressiveness. More detailed investigation is required to clarify the significance of these modifications of SAM68.

SAM68 is known to play important roles in the regulation of mRNA processing, signal transduction, gene transcription, and DNA repair [33,54], and emerging evidence links SAM68 to pathogenic mechanisms, including cell proliferation, apoptosis, invasion, and metastasis in various human cancers [55,56,57,58]. Previous studies and our data here agree in that upregulation of SAM68 expression is associated with lung cancer progression and poor patient outcome [37,39,40,41]. However, the molecular mechanisms in which SAM68 participates in the pathogenesis of lung cancer still await elucidation. It has been suggested that SAM68 can promote the proliferation of NSCLC cells by activating the Wnt/β-catenin pathway [40]. A recent report indicates that SAM68 promotes tumorigenesis and cancer metabolic programming in lung adenocarcinoma cells by regulating RNA splicing to increase the formation of oncogenic pyruvate kinase PKM2; the C-terminal region (aa 351-443) of SAM68 is a key functional domain in this regulation [37]. Here, we have added that *O*-GlcNAcylation of SAM68 in the *N*-terminal region has functional significance in lung cancer cell migration and invasion. Together, findings obtained by us and others establish that SAM68 plays a pivotal in lung cancer pathogenesis, and pursuing the understanding of its molecular regulation should help in guiding the development of novel SAM68-based diagnostic or therapeutic reagents.

## 5. Conclusions

We obtained a proteomic profile containing 264 candidates for invasiveness-related *O*-GlcNAcylated proteins in lung adenocarcinoma cells and further characterized SAM68 concerning its *O*-GlcNAcylation and involvement in cancer cell aggressiveness. Our data suggest that *O*-GlcNAcylation sites in the *N*-terminal region are important for the function of SAM68 in regulating cancer cell migration and invasion and that concomitant high expression of SAM68 and OGT in lung adenocarcinoma tissues is associated with poor patient outcome. These findings highlight the potential of invasiveness-associated *O*-GlcNAcylated proteins as novel biomarkers for lung cancer prognosis. Further understanding of the functional consequence of differential *O*-GlcNAcylation of proteins identified in this study may provide new insights into the mechanisms of lung cancer progression.

## Data Availability

The data presented in this report are available from the corresponding authors upon reasonable request.

## Supplementary Materials

The following are available online at https://www.mdpi.com/article/10.3390/cancers14010243/s1, Figure S1: Enrichment of nuclear/cytosolic *O*-GlcNAcylated proteins, Figure S2: Gene ontology analysis, Figure S3: The actin cytoskeleton signaling pathway, Figure S4: Identification of *O*-GlcNAcylation sites by MS/MS analysis, Figure S5: Comparison of *O*-GlcNAcylation levels of wild-type and mutant SAM68, Figure S6: Kaplan-Meier analysis of the overall survival of patients with lung adenocarcinoma, Figure S7: Full Western blot for Figure 1B, Figure S8: Full Western blot for Figure 2C,D, Figure S9: Full Western blot for Figure 3A–C, Figure S10: Full Western blot for Figure 4B, Figure S11: Full Western blot for Figure 4C, Figure S12: Full Western blot for Figure 4D, Figure S13: Full Western blot for Figure 5A and 5D, Figure S14: Full Western blot for Figure S1, Figure S15: Full Western blot for Figure S5, Table S1: Differential nuclear WGA-bound proteins, Table S2: Differential cytosolic WGA-bound proteins, Table S3: Significant network functions, Table S4: Significant canonical pathways, Table S5: Significant molecular and cellular functions, Table S6: Association of SAM68 expression with lung adenocarcinoma stage.
