# Supplementary material for: Comparative O-GlcNAc Proteomic Analysis Reveals a Role of O-GlcNAcylated SAM68 in Lung Cancer Aggressiveness
Source: Cancers (Basel). 2022 Jan 4;14(1):243. doi: 10.3390/cancers14010243 (PMC8749979; doi:10.3390/cancers14010243)
Supplement: Supplementary file 1 [file cancers-14-00243-s001.zip › cancers-1481759-supplementary.pdf]

Supplementary Materials

Comparative *O*-GlcNAc Proteomic Analysis Reveals a Role of *O*-GlcNAcylated SAM68 in Lung Cancer Aggressiveness

Chia-Hung Lin, Chen-Chung Liao, Shu-Ying Wang, Chia-Yi Peng, Yi-Chen Yeh, Mei-Yu Chen and Teh-Ying Chou

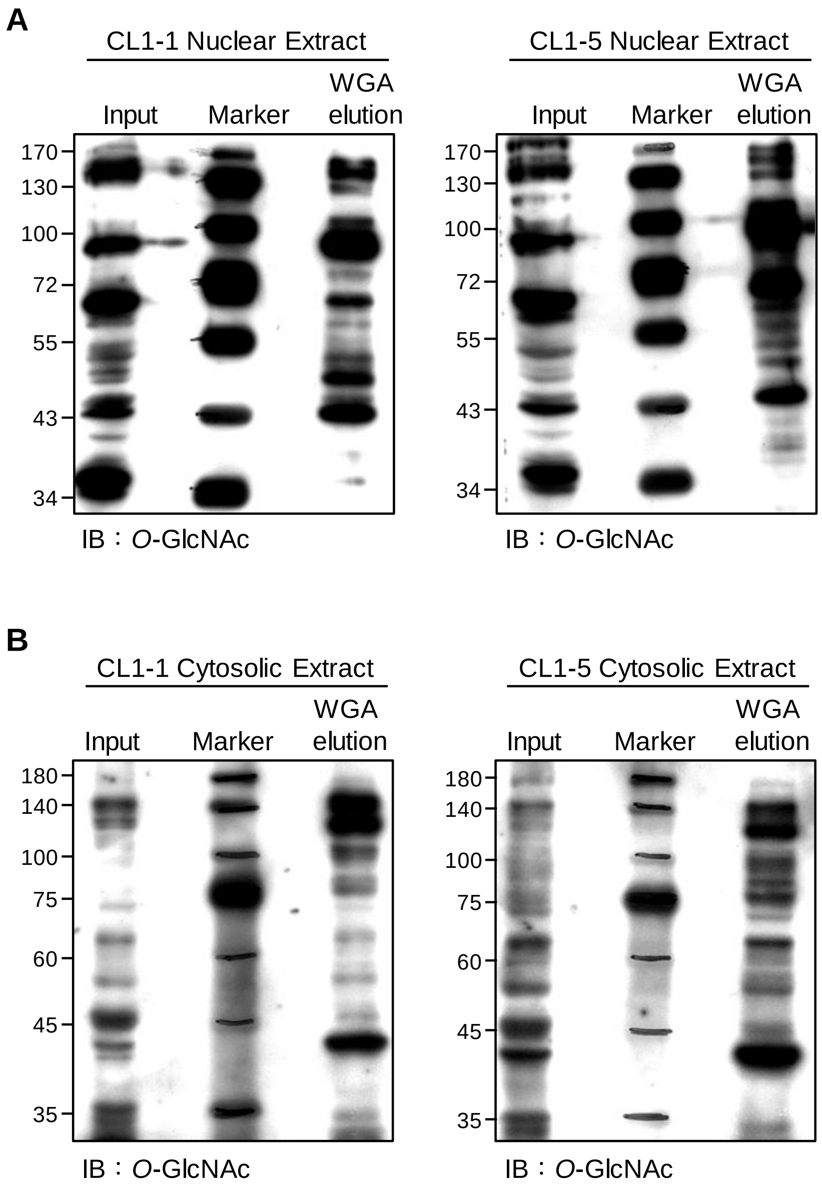

Figure S1. Enrichment of nuclear/cytosolic O-GlcNAcylated proteins. Nuclear (**A**) and cytosolic (**B**) extracts from CL1-1 and CL1-5 cells were subjected to GlcNAc-binding lectin-based affinity chromatography using resin-bound wheat germ agglutinin (WGA). Proteins in the extracts (input) and the WGA elution fractions were separated by SDS-PAGE and were immunoblotted (IB) using *O*-GlcNAc antibodies. Each input sample contained 100 µg of proteins, and each WGA elution sample contained 5 µg of proteins. Detailed information about the Western blotting can be found in Figure S14.

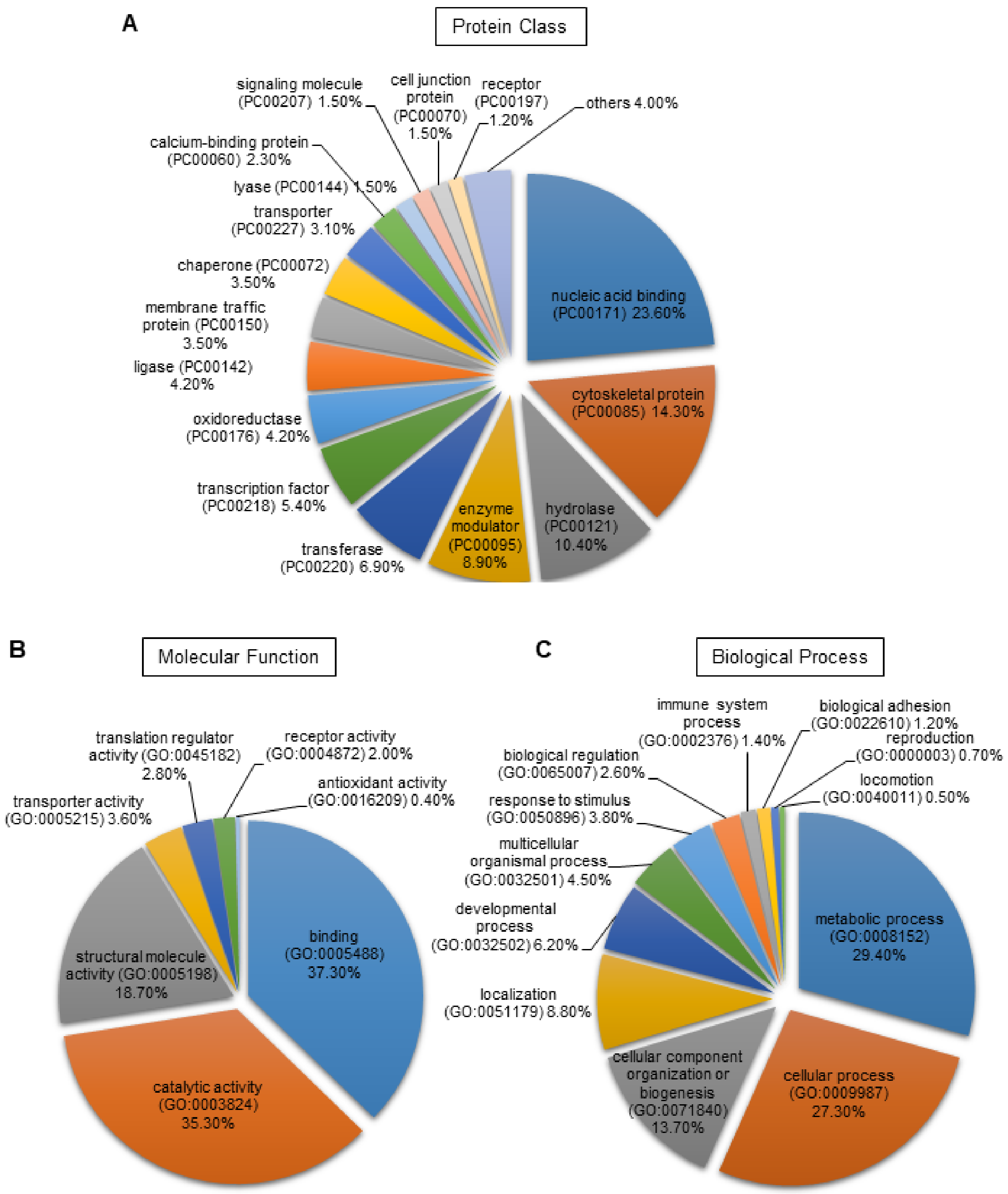

Figure S2. Gene ontology analysis. Differential WGA-bound nuclear/cytosolic glycoproteins between CL1 and CL5 samples were classified according to their protein classes (**A**), molecular functions (**B**), and biological processes (**C**) using the PANTHER classification system (http://www.pantherdb.org/, accessed on 13 Feb 2019). WGA, wheat germ agglutinin; PANTHER, Protein ANalysis THrough Evolutionary Relationships.

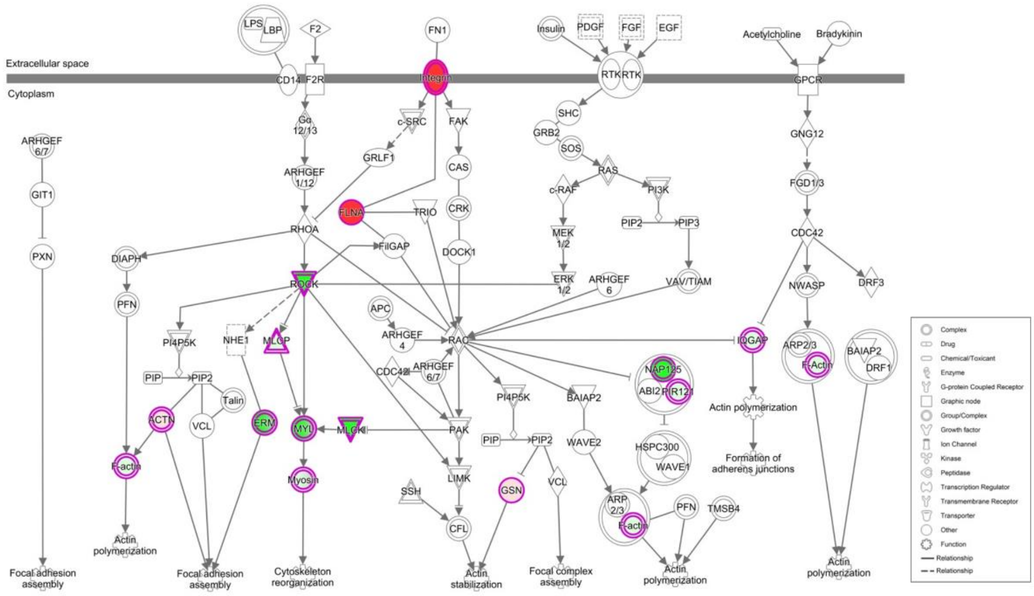

Figure S3. The actin cytoskeleton signaling pathway. Ingenuity Pathway Analysis of the differential wheat germ agglutinin (WGA)-bound nuclear/cytosolic glycoproteins identified this pathway as the top canonical pathway. Proteins with purple outlines are differential WGA-bound glycoproteins identified in this study; those in red and green shades are up- and down-regulated, respectively, in CL1-5 cells relative to CL1-1 cells. Proteins in white-colored symbols are not in our list of differential WGA-bound glycoproteins but were incorporated into the network based on the Ingenuity database.

A

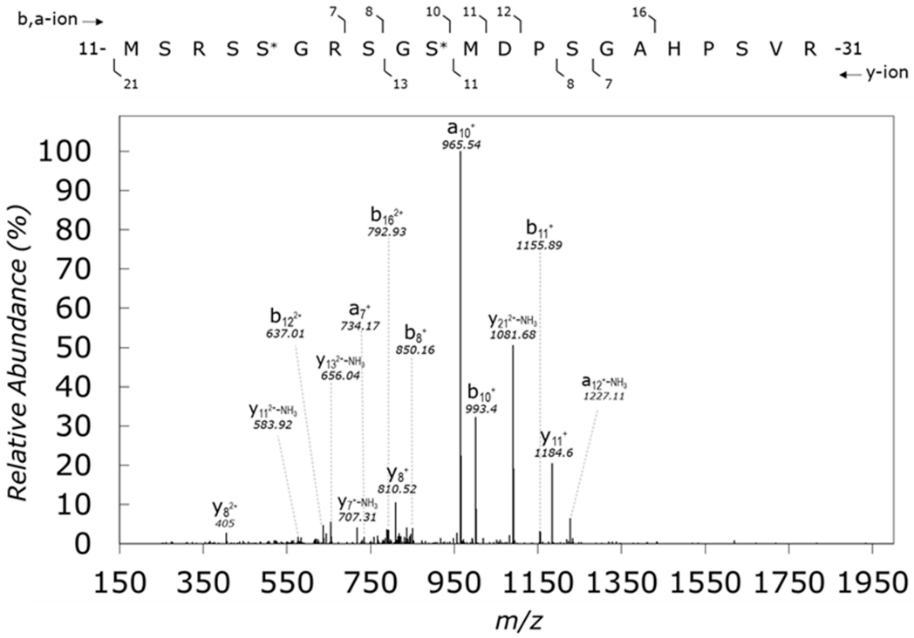

B

Site	Peptide sequence	Immunoprecipitated	WGA-bound
S12	MSRSSGRSGSMDPSGAHPSVR	–	*O*-GlcNAc
S15	MSRSSGRSGSMDPSGAHPSVR	*O*-GlcNAc	–
S18	MSRSSGRSGSMDPSGAHPSVR	–	*O*-GlcNAc
S20	MSRSSGRSGSMDPSGAHPSVR	*O*-GlcNAc	*O*-GlcNAc
S24	SSGRSGSMDPSGAHPSVR	–	*O*-GlcNAc
T183	FNFVGKILGPQGNTIKR	–	*O*-GlcNAc
S202	LQEETGAKISVLGKGSMR	–	*O*-GlcNAc
T317	GTPVRGAITRGATVTR	–	*O*-GlcNAc
T324	GAITRGATVTRGVPPPPTVR	–	*O*-GlcNAc
T330	GATVTRGVPPPPTVRGAPAPR	–	*O*-GlcNAc
S422	PSLKAPPARPVK	*O*-GlcNAc	–

Figure S4. Identification of *O*-GlcNAcylation sites by MS/MS analysis. (**A**) A representative MS/MS spectrum of the peptide with a sequence of 11-MSRSS*GRSGS*MDPSGAHPSVR-31 is shown as an example for *O*-GlcNAcylation site identification; *, *O*-GlcNAcylated Ser residue. (**B**) A summary of *O*-GlcNAcylation sites revealed by results from multiple experiments of MS/MS analysis on wheat germ agglutinin (WGA)-bound or immunoprecipitated SAM68 samples. Underlined are identified *O*-GlcNAcylated residues.

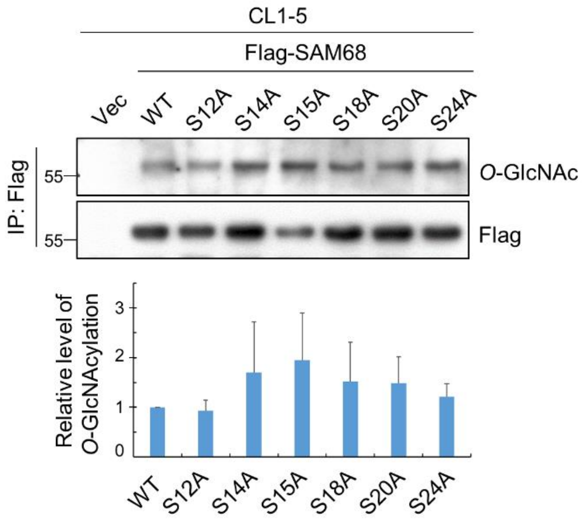

Figure S5. Comparison of *O*-GlcNAcylation levels of wild-type and mutant SAM68. Whole-cell lysates from CL1-5 cells transfected with the control vector (Vec) or an expression construct of wild-type (WT) or single-site mutant of SAM68 were subjected to immunoprecipitation (IP) and Western analysis. Representative Western results and quantitative data (means ± SD) from three independent experiments are shown. The relative level of *O*-GlcNAcylation for each mutant was calculated by normalizing the *O*-GlcNAc/Flag Western signal ratio to that of the WT samples. Detailed information about the Western blotting can be found in Figure S15.

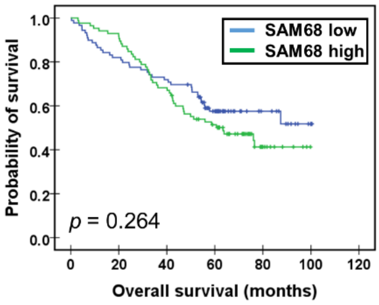

Figure S6. Kaplan-Meier analysis of the overall survival of patients with lung adenocarcinoma. Patients (n = 174) were categorized into high and low SAM68 expression groups according to the medium IHC score. p-values were derived from the log-rank test.

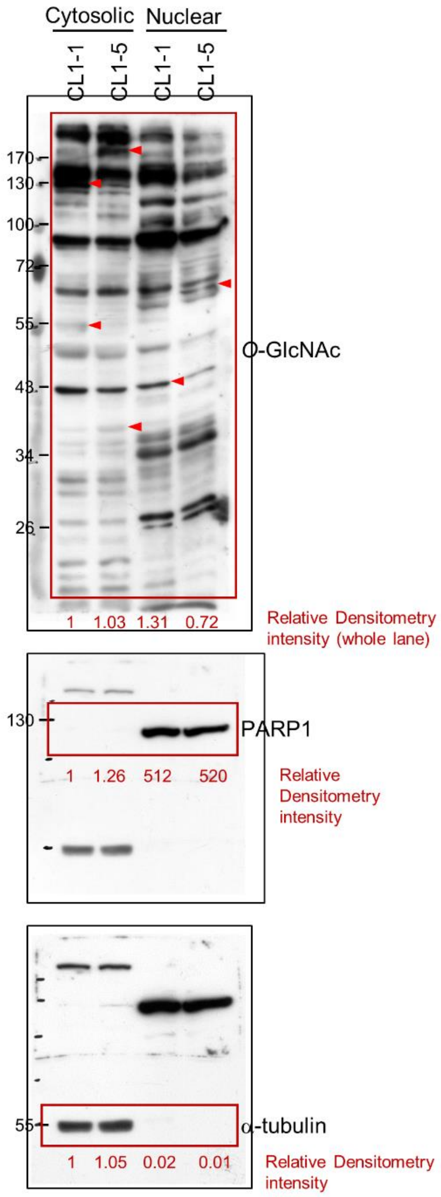

Figure S7. Full Western blot for Figure 1B.

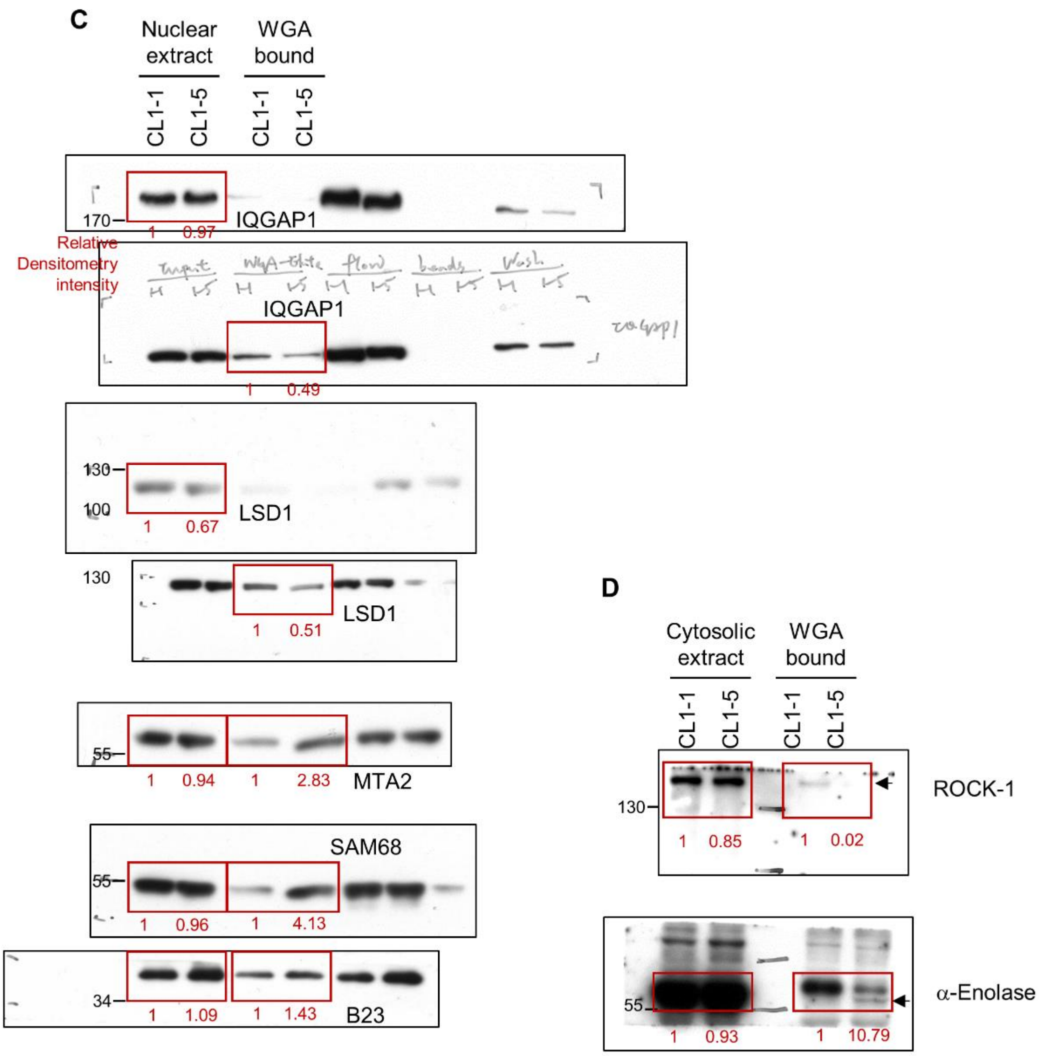

Figure S8. Full Western blot for Figure 2C and 2D. WGA, wheat germ agglutinin.

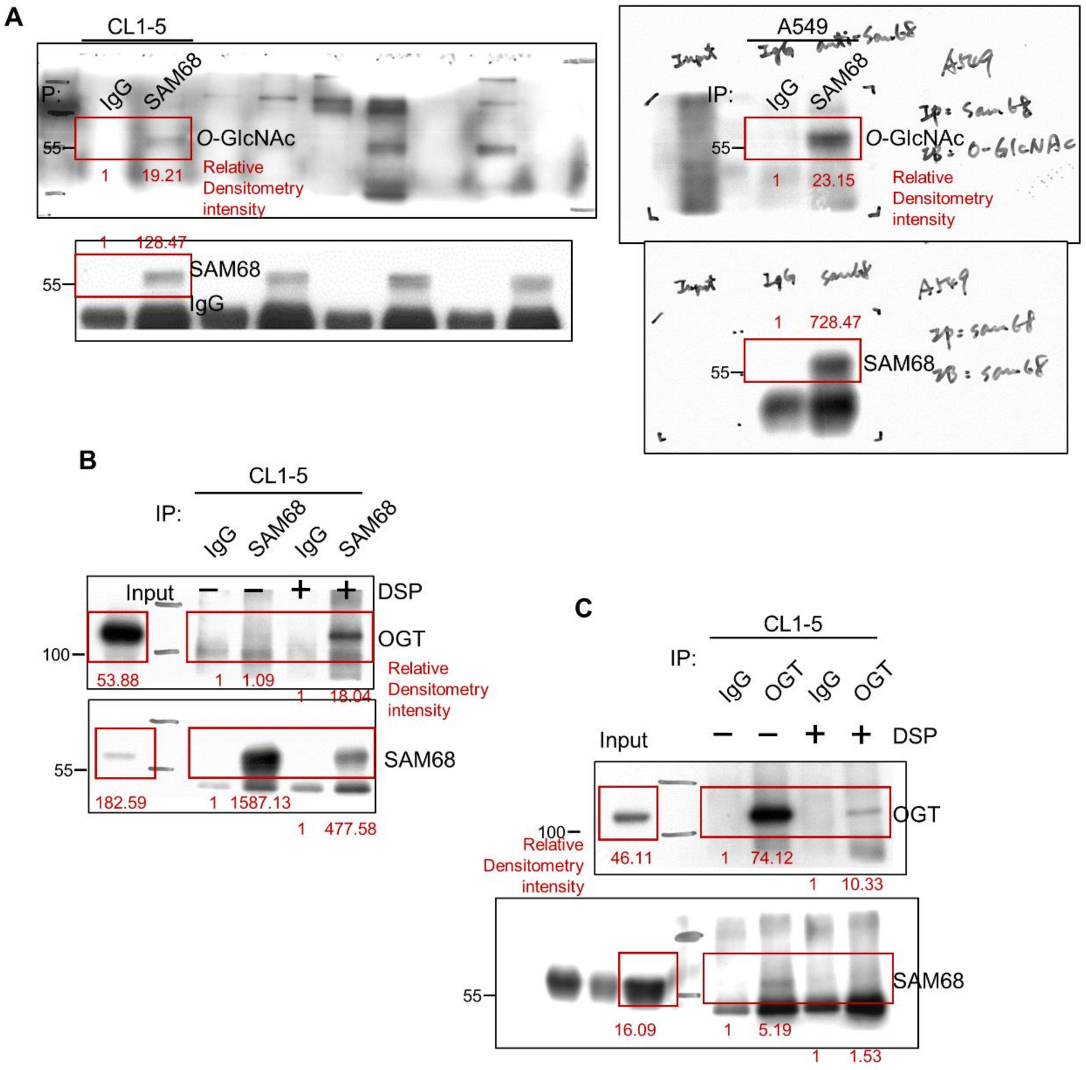

Figure S9. Full Western blot for Figure 3A–C. OGT, *O*-GlcNAc transferase

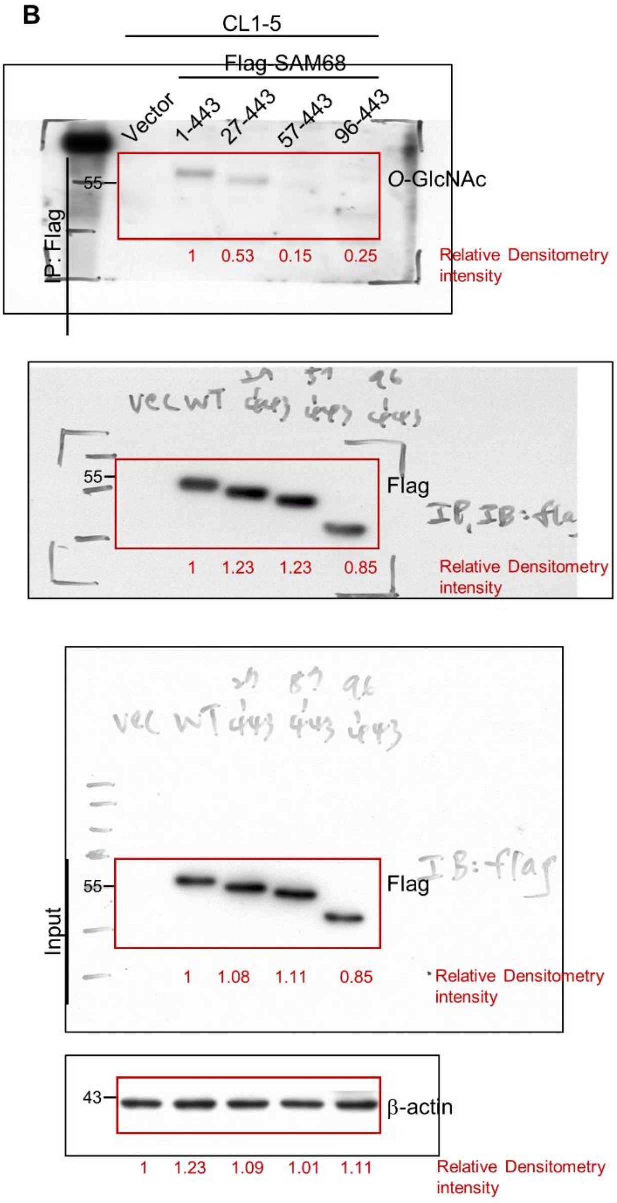

Figure S10. Full Western blot for Figure 4B.

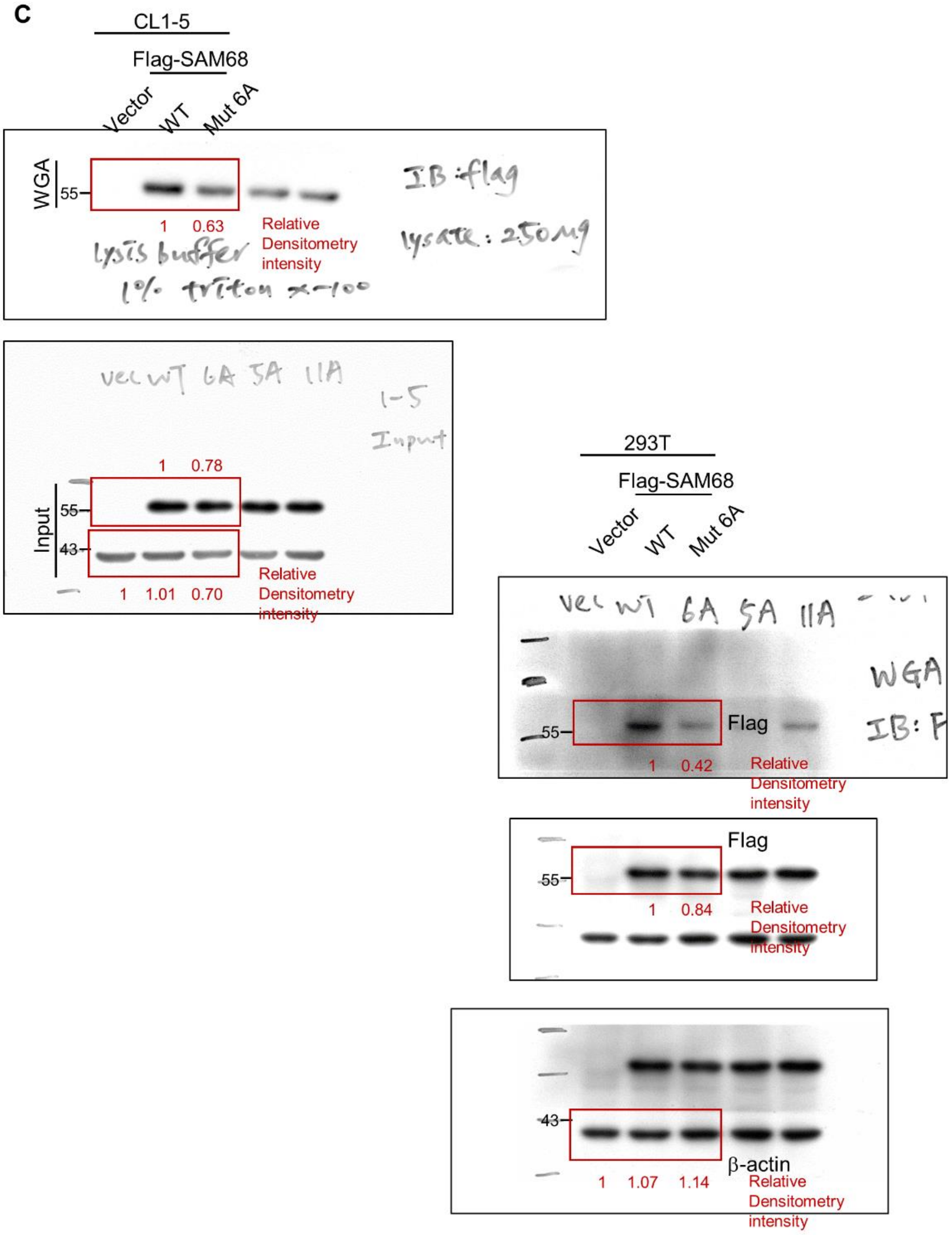

Figure S11. Full Western blot for Figure 4C.

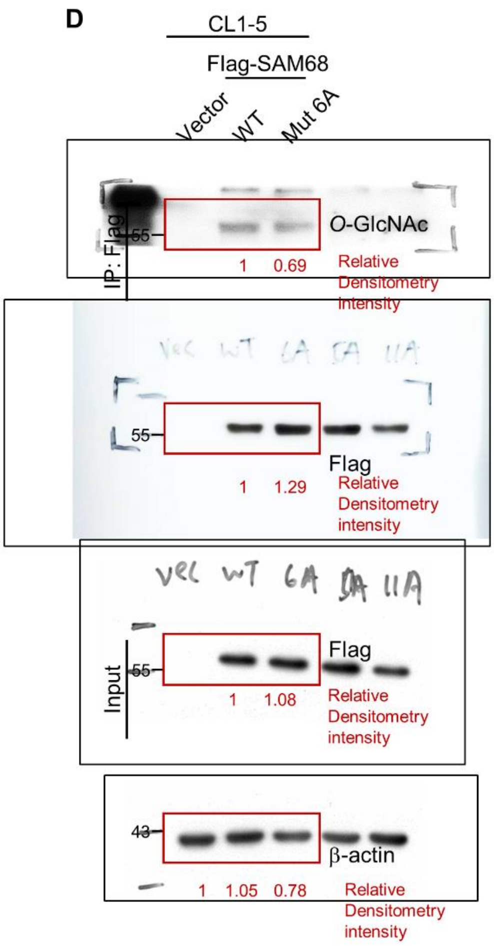

Figure S12. Full Western blot for Figure 4D.

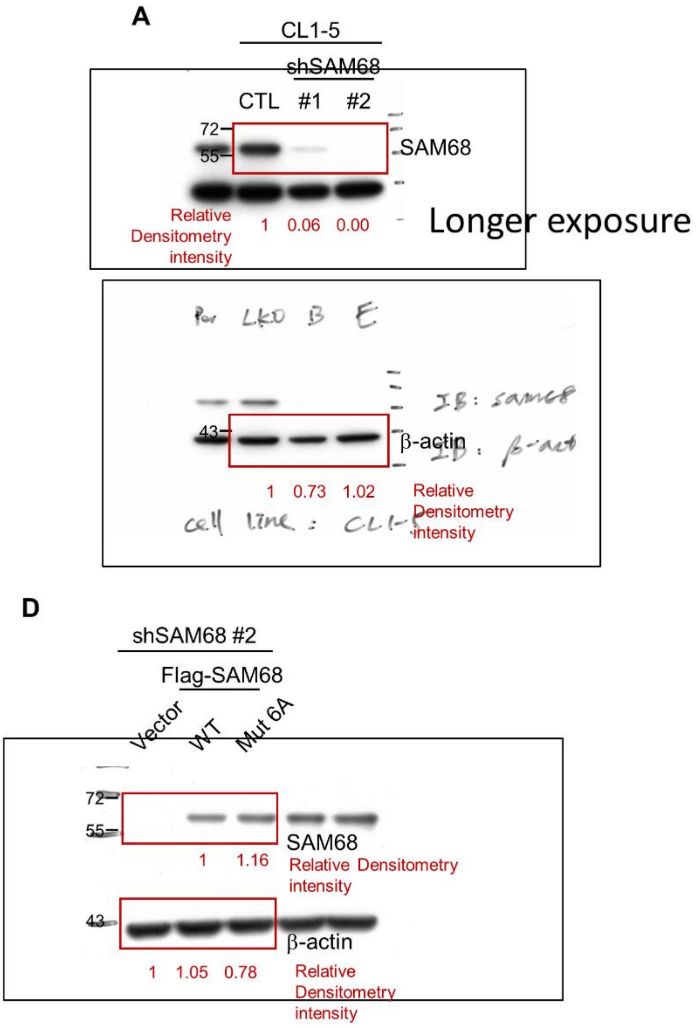

Figure S13. Full Western blot for Figure 5A and 5D.

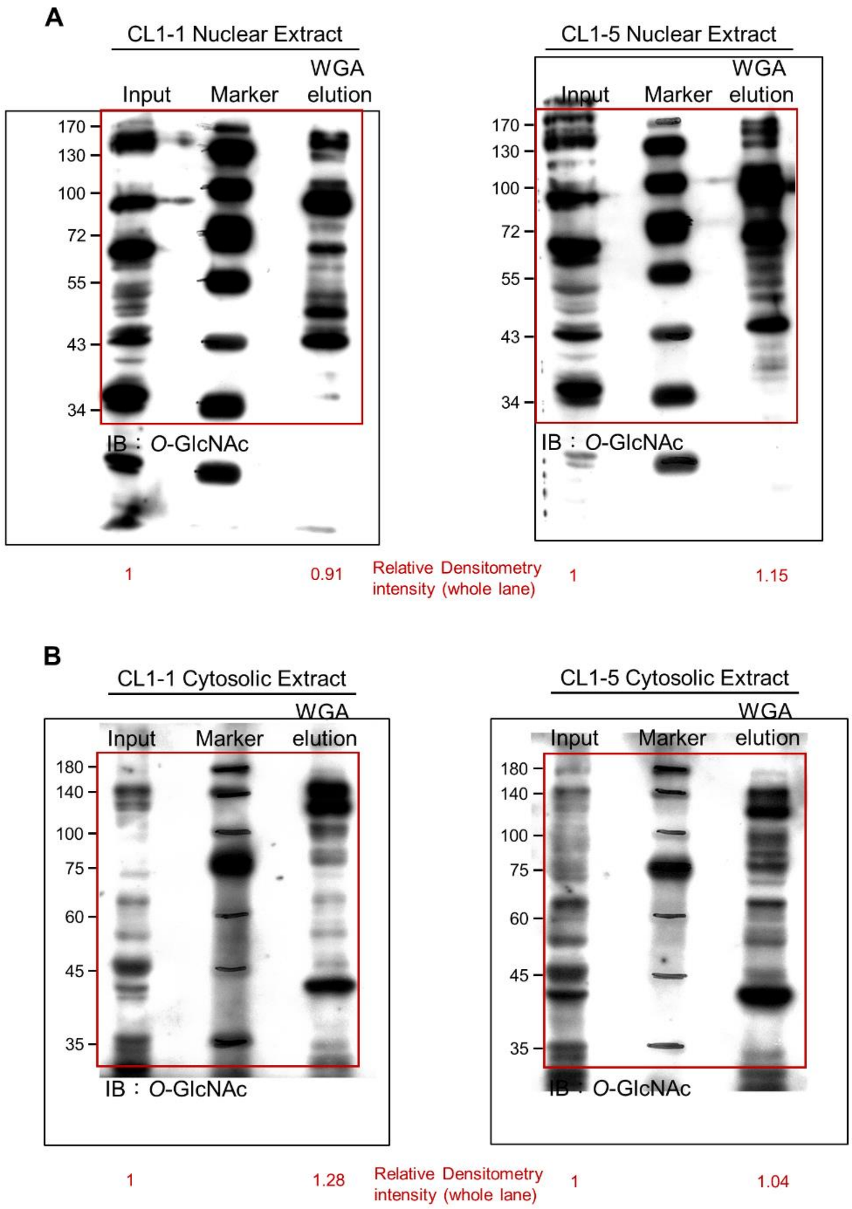

Figure S14. Full Western blot for Figure S1.

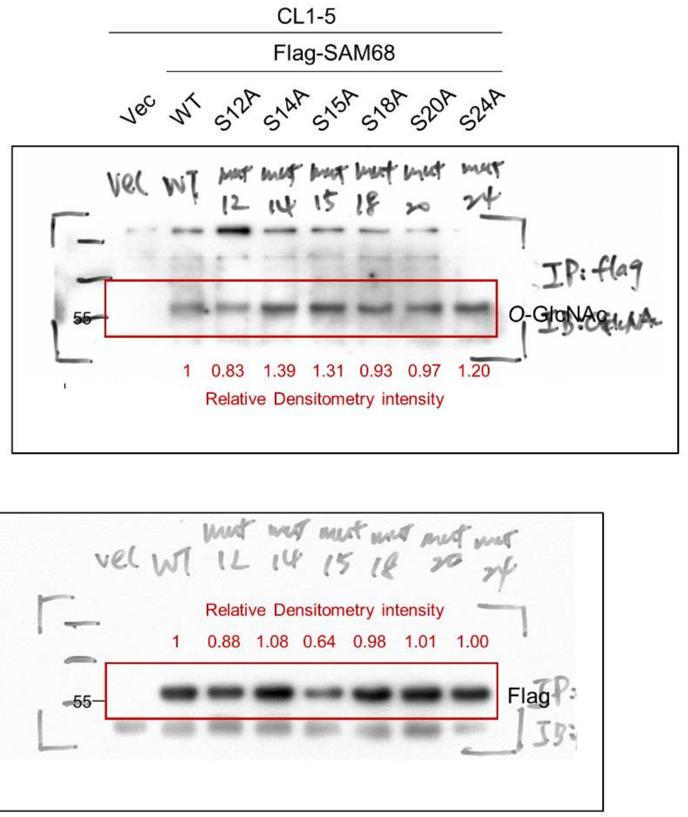

Figure S15. Full Western blot for Figure S5.

Table S1. Differential WGA-bound glycoproteins identified in nuclear extracts from lung adenocarinoma cell lines CL1-1 and CL1-5.

Protein ID	Symbol	Protein Name	Normalized Spectral Counts (Mean ± SE) [1]		*p*-Value [2]	Fold Change	Listed in the O-GlcNAc Database [3]
			In CL1-1	In CL1-5			
		Down-regulated in CL1-5					
E7EVA0	E7EVA0	Microtubule-associated protein	23.51 ± 6.02	1.03 ± 1.03	0.021	↓22.90	no
P35580	MYH10	Myosin-10	155.97 ± 16.06	13.93 ± 5.70	0.001	↓11.20	yes
P12532	KCRU	Creatine kinase U-type, mitochondrial	7.74 ± 1.19	0.70 ± 0.70	0.007	↓11.01	yes
P02786	TFR1	Transferrin receptor protein 1	5.01 ± 0.59	0.51 ± 0.51	0.005	↓9.75	yes
H3BPE1	H3BPE1	Microtubule-actin cross-linking factor 1, isoforms 1/2/3/5	9.46 ± 0.94	1.20 ± 1.20	0.006	↓7.89	yes
Q7L576	CYFP1	Cytoplasmic FMR1-interacting protein 1	8.96 ± 1.86	1.32 ± 1.32	0.029	↓6.78	yes
P19801	AOC1	Amiloride-sensitive amine oxidase [copper-containing]	4.33 ± 0.28	0.69 ± 0.69	0.008	↓6.32	no
Q06203	PUR1	Amidophosphoribosyltransferase	3.17 ± 0.43	0.51 ± 0.51	0.016	↓6.17	yes
A0A087WWU8	A0A087WWU8	Tropomyosin alpha-3 chain	20.43 ± 2.24	3.54 ± 1.78	0.004	↓5.78	yes
P35749	MYH11	Myosin-11	37.60 ± 2.06	7.07 ± 2.72	0.001	↓5.32	yes
P78347	GTF2I	General transcription factor II-I	4.49 ± 0.43	0.99 ± 0.99	0.032	↓4.53	yes
Q96ST3	SIN3A	Paired amphipathic helix protein Sin3a	4.54 ± 0.86	1.02 ± 0.51	0.025	↓4.44	yes
P09874	PARP1	Poly [ADP-ribose] polymerase 1	5.00 ± 0.44	1.19 ± 0.61	0.007	↓4.21	yes
P12277	KCRB	Creatine kinase B-type	25.99 ± 2.47	6.22 ± 2.65	0.006	↓4.18	yes
O60341	KDM1A	Lysine-specific histone demethylase 1A	6.24 ± 1.14	1.53 ± 0.77	0.027	↓4.08	yes
Q8NE71	ABCF1	ATP-binding cassette sub-family F member 1	2.65 ± 0.26	0.66 ± 0.66	0.049	↓4.01	yes
Q16568	CART	Cocaine- and amphetamine-regulated transcript protein	2.00 ± 0.06	0.51 ± 0.51	0.045	↓3.90	no
Q7Z406	MYH14	Myosin-14	24.04 ± 2.58	7.91 ± 2.32	0.010	↓3.04	yes
P55884	EIF3B	Eukaryotic translation initiation factor 3 subunit B	6.11 ± 0.94	2.04 ± 1.05	0.044	↓3.00	yes
Q04917	1433F	14-3-3 protein eta	11.19 ± 1.03	3.74 ± 0.59	0.003	↓2.99	yes
Q15642	CIP4	Cdc42-interacting protein 4	7.15 ± 0.41	2.56 ± 0.28	0.001	↓2.80	yes
Q9NYU2	UGGG1	UDP-glucose:glycoprotein glucosyltransferase 1	17.80 ± 1.58	6.46 ± 0.89	0.003	↓2.75	yes
P35579	MYH9	Myosin-9	285.90 ± 25.16	104.71 ± 18.50	0.004	↓2.73	yes
P67936	TPM4	Tropomyosin alpha-4 chain	17.63 ± 2.69	6.66 ± 0.27	0.015	↓2.65	yes
P46940	IQGA1	Ras GTPase-activating-like protein IQGAP1	75.84 ± 6.65	34.35 ± 5.51	0.009	↓2.21	yes
Q9Y4L1	HYOU1	Hypoxia up-regulated protein 1	11.64 ± 1.25	5.29 ± 0.44	0.009	↓2.20	yes
Q6S8J3	POTEE	POTE ankyrin domain family member E	41.86 ± 3.77	19.18 ± 4.09	0.015	↓2.18	yes
P22314	UBA1	Ubiquitin-like modifier-activating enzyme 1	36.53 ± 2.91	16.98 ± 2.72	0.008	↓2.15	yes
P61981	1433G	14-3-3 protein gamma	21.05 ± 2.90	10.01 ± 1.87	0.033	↓2.10	yes
Q14697	GANAB	Neutral alpha-glucosidase AB	40.04 ± 0.65	20.45 ± 2.27	0.001	↓1.96	yes
Q15691	MARE1	Microtubule-associated protein RP/EB family member 1	23.57 ± 2.35	13.29 ± 0.55	0.013	↓1.77	yes
P34932	HSP74	70-kDa Heat-shock protein 4	45.24 ± 1.14	26.38 ± 3.04	0.004	↓1.72	yes
P49321	NASP	Nuclear autoantigenic sperm protein	16.74 ± 1.81	10.04 ± 1.35	0.041	↓1.67	yes
V9HVZ7	V9HVZ7	Epididymis luminal protein 176	73.03 ± 3.75	45.49 ± 8.25	0.038	↓1.61	no
O14776	TCRG1	Transcription elongation regulator 1	45.05 ± 2.81	29.02 ± 4.82	0.045	↓1.55	yes
Q562R1	ACTBL	Beta-actin-like protein 2	31.54 ± 0.55	20.56 ± 3.45	0.035	↓1.53	yes
Q01970	PLCB3	1-phosphatidylinositol 4,5-bisphosphate phosphodiesterase beta-3	2.17 ± 0.42	N.D.	0.007	-	yes

P08195	4F2	4F2 cell-surface antigen heavy chain	1.84 ± 0.20	N.D.	0.001	-	yes
P17050	NAGAB	Alpha-N-acetylgalactosaminidase	2.51 ± 0.08	N.D.	0.000	-	yes
P04083	ANXA1	Annexin A1	14.83 ± 3.17	N.D.	0.010	-	yes
Q8IZY2	ABCA7	ATP-binding cassette sub-family A member 7	1.83 ± 0.12	N.D.	0.000	-	yes
Q9P2I0	CPSF2	Cleavage and polyadenylation specificity factor subunit 2	3.33 ± 0.56	N.D.	0.004	-	yes
P43246	MSH2	DNA mismatch repair protein Msh2	4.66 ± 1.42	N.D.	0.031	-	yes
P23434	GCSH	Glycine cleavage system H protein, mitochondrial	3.54 ± 0.84	N.D.	0.014	-	yes
P41250	SYG	Glycine--tRNA ligase	4.66 ± 1.15	N.D.	0.015	-	yes
Q8WXG9	GPR98	G-protein coupled receptor 98	3.01 ± 0.09	N.D.	0.000	-	yes
P04792	HSPB1	Heat-shock protein beta-1	3.17 ± 0.71	N.D.	0.011	-	yes
A8K9J7	A8K9J7	Histone H2B	2.71 ± 0.77	N.D.	0.024	-	yes
D3DS63	D3DS63	Importin 4, isoform CRA_f	4.30 ± 0.56	N.D.	0.002	-	no
P05556	ITB1	Integrin beta-1	2.80 ± 0.60	N.D.	0.010	-	yes
P19105	ML12A	Myosin regulatory light chain 12A	6.93 ± 1.58	N.D.	0.012	-	yes
O14950	ML12B	Myosin regulatory light chain 12B	6.93 ± 1.58	N.D.	0.012	-	no
Q9Y2A7	NCKP1	Nck-associated protein 1	2.64 ± 0.67	N.D.	0.017	-	yes
H0YN18	H0YN18	Proteasome subunit alpha type-4	3.15 ± 0.37	N.D.	0.001	-	yes
Q04837	SSBP	Single-stranded DNA-binding protein, mitochondrial	3.86 ± 0.73	N.D.	0.006	-	yes
Q14683	SMC1A	Structural maintenance of chromosomes protein 1A	3.64 ± 0.64	N.D.	0.005	-	yes
Q6IMI6	ST1C3	Sulfotransferase 1C3	2.18 ± 0.24	N.D.	0.001	-	no
A8K3C3	A8K3C3	T-complex protein 1 subunit delta	2.84 ± 0.22	N.D.	0.000	-	yes
Q99598	TSNAX	Translin-associated protein X	1.99 ± 0.24	N.D.	0.001	-	yes
B7Z596	B7Z596	Tropomyosin alpha-1 chain	10.16 ± 1.86	N.D.	0.006	-	yes
Q9Y4Z0	LSM4	U6 snRNA-associated Sm-like protein LSm4	3.39 ± 0.96	N.D.	0.024	-	yes
Q9UMX0	UBQL1	Ubiquilin-1	5.11 ± 1.38	N.D.	0.021	-	yes
P54725	RD23A	UV excision repair protein RAD23 homolog A	2.36 ± 0.42	N.D.	0.005	-	yes
		Up-regulated in CL1-5					
Q6IN85	P4R3A	Serine/threonine-protein phosphatase 4 regulatory subunit 3A	0.49 ± 0.48	7.65 ± 2.52	0.049	↑15.78	yes
P07910	HNRPC	Heterogeneous nuclear ribonucleoproteins C1/C2	0.71 ± 0.71	11.18 ± 2.67	0.019	↑15.77	yes
P07355	ANXA2	Annexin A2	2.89 ± 1.47	33.55 ± 3.09	0.001	↑11.60	yes
P08758	ANXA5	Annexin A5	2.07 ± 1.23	16.18 ± 1.09	0.001	↑7.83	yes
O43670	ZN207	BUB3-interacting and GLEBS motif-containing protein ZNF207	0.49 ± 0.48	3.07 ± 0.28	0.010	↑6.33	yes
Q96DI7	SNR40	U5 small nuclear ribonucleoprotein 40 kDa protein	0.97 ± 0.97	5.99 ± 1.10	0.027	↑6.18	yes
O00232	PSD12	26S proteasome non-ATPase regulatory subunit 12	0.49 ± 0.49	2.72 ± 0.60	0.044	↑5.58	yes
Q9H0D6	XRN2	5'-3' exoribonuclease 2	1.52 ± 0.77	7.80 ± 1.36	0.016	↑5.13	yes
Q9BUT1	BDH2	3-hydroxybutyrate dehydrogenase type 2	0.97 ± 0.97	4.94 ± 0.90	0.040	↑5.10	no
O94776	MTA2	Metastasis-associated protein MTA2	1.34 ± 0.71	6.80 ± 0.79	0.007	↑5.06	yes
Q14997	PSME4	Proteasome activator complex subunit 4	1.95 ± 1.13	9.35 ± 2.03	0.033	↑4.81	yes
Q9H2G2	SLK	STE20-like serine/threonine-protein kinase	0.65 ± 0.65	2.74 ± 0.38	0.050	↑4.23	yes
Q12905	ILF2	Interleukin enhancer-binding factor 2	5.11 ± 2.08	19.82 ± 1.23	0.004	↑3.88	yes
O14979	HNRDL	Heterogeneous nuclear ribonucleoprotein D-like	3.66 ± 1.15	12.41 ± 1.50	0.010	↑3.39	yes
Q99436	PSB7	Proteasome subunit beta type-7	1.68 ± 0.87	5.64 ± 0.65	0.022	↑3.35	yes
Q15149	PLEC	Plectin	26.31 ± 3.07	84.28 ± 13.73	0.015	↑3.20	yes

P10809	CH60	60-kDa Heat-shock protein, mitochondrial	13.60 ± 1.96	39.35 ± 3.53	0.003	↑2.89	yes
P35637	FUS	RNA-binding protein FUS	2.92 ± 1.85	8.37 ± 0.31	0.043	↑2.87	yes
P08133	ANXA6	Annexin A6	11.05 ± 5.74	31.67 ± 3.18	0.035	↑2.87	yes
O60568	PLOD3	Procollagen-lysine,2-oxoglutarate 5-dioxygenase 3	3.48 ± 0.70	9.79 ± 1.97	0.039	↑2.81	yes
Q9UMS4	PRP19	Pre-mRNA-processing factor 19	7.84 ± 0.28	18.93 ± 1.39	0.001	↑2.42	yes
P60900	PSA6	Proteasome subunit alpha type-6	3.39 ± 1.00	8.17 ± 1.38	0.049	↑2.41	yes
O43684	BUB3	Mitotic checkpoint protein BUB3	4.81 ± 1.51	11.55 ± 1.62	0.038	↑2.40	yes
O43390	HNRPR	Heterogeneous nuclear ribonucleoprotein R	14.48 ± 0.52	34.46 ± 5.17	0.018	↑2.38	yes
A0A087WZV1	A0A087WZV1	Heterogeneous nuclear ribonucleoprotein A/B	9.40 ± 1.87	22.25 ± 3.26	0.027	↑2.37	yes
P17096	HMGA1	High mobility group protein HMG-I/HMG-Y	4.89 ± 1.01	11.46 ± 1.17	0.013	↑2.34	yes
O43660	PLRG1	Pleiotropic regulator 1	5.78 ± 2.07	12.80 ± 0.07	0.028	↑2.22	yes
P55072	TERA	Transitional endoplasmic reticulum ATPase	21.21 ± 2.37	46.92 ± 5.62	0.014	↑2.21	yes
P12956	XRCC6	X-ray repair cross-complementing protein 6	3.02 ± 0.38	6.66 ± 1.04	0.030	↑2.21	yes
P25786	PSA1	Proteasome subunit alpha type-1	5.25 ± 1.36	11.21 ± 1.60	0.047	↑2.14	yes
P06748	NPM	Nucleophosmin	4.00 ± 0.70	8.36 ± 1.36	0.046	↑2.09	yes
Q8N684	CPSF7	Cleavage and polyadenylation specificity factor subunit 7	3.18 ± 0.39	6.62 ± 1.17	0.049	↑2.08	yes
Q01844	EWS	RNA-binding protein EWS	6.60 ± 1.20	13.50 ± 0.56	0.007	↑2.05	yes
P25788	PSA3	Proteasome subunit alpha type-3	3.39 ± 1.00	6.65 ± 0.25	0.034	↑1.96	yes
P31942	HNRH3	Heterogeneous nuclear ribonucleoprotein H3	13.49 ± 1.11	26.32 ± 3.98	0.036	↑1.95	yes
P08865	RSSA	40S ribosomal protein SA	10.89 ± 1.56	19.95 ± 0.53	0.005	↑1.83	yes
P61978	HNRPK	Heterogeneous nuclear ribonucleoprotein K	41.58 ± 1.70	72.76 ± 2.65	0.001	↑1.75	yes
Q13547	HDAC1	Histone deacetylase 1	6.35 ± 0.51	10.92 ± 0.34	0.002	↑1.72	yes
Q13409	DC1I2	Cytoplasmic dynein 1 intermediate chain 2	1.84 ± 0.20	3.08 ± 0.35	0.037	↑1.67	yes
Q07666	KHDR1	KH domain-containing, RNA-binding, signal transduction-associated protein 1	14.00 ± 2.94	23.38 ± 1.68	0.047	↑1.67	yes
Q13283	G3BP1	Ras GTPase-activating protein-binding protein 1	6.63 ± 0.71	10.93 ± 0.38	0.006	↑1.65	yes
G3V1C3	G3V1C3	Apoptosis inhibitor 5	33.06 ± 2.01	52.43 ± 1.57	0.002	↑1.59	yes
Q13765	NACA	Nascent polypeptide-associated complex subunit alpha	11.93 ± 1.64	18.25 ± 1.23	0.037	↑1.53	yes
P43686	PRS6B	26S protease regulatory subunit 6B	N.D.	3.57 ± 0.45	0.001	-	yes
P62195	PRS8	26S protease regulatory subunit 8	N.D.	1.87 ± 0.31	0.004	-	yes
O00487	PSDE	26S proteasome non-ATPase regulatory subunit 14	N.D.	2.55 ± 0.58	0.012	-	yes
P55036	PSMD4	26S proteasome non-ATPase regulatory subunit 4	N.D.	3.61 ± 0.84	0.012	-	yes
P15144	AMPN	Aminopeptidase N	N.D.	8.91 ± 0.99	0.001	-	yes
E7ETM0	E7ETM0	Casein kinase I isoform alpha	N.D.	3.76 ± 0.35	0.000	-	yes
P36957	ODO2	Dihydrolipoyllysine-residue succinyltransferase component of 2-oxoglutarate dehydrogenase complex, mitochondrial	N.D.	4.75 ± 1.15	0.014	-	yes
O15160	RPAC1	DNA-directed RNA polymerases I and III subunit RPAC1	N.D.	2.05 ± 0.29	0.002	-	yes
P78406	RAE1L	mRNA export factor	N.D.	5.33 ± 1.90	0.048	-	yes
P49790	NU153	Nuclear pore complex protein Nup153	N.D.	3.95 ± 0.96	0.015	-	yes
Q8NC51	PAIRB	Plasminogen activator inhibitor 1 RNA-binding protein	N.D.	7.70 ± 1.11	0.002	-	yes
P0CG47	UBB	Polyubiquitin-B	N.D.	4.80 ± 0.67	0.002	-	yes
P0CG48	UBC	Polyubiquitin-C	N.D.	4.80 ± 0.67	0.002	-	yes
Q8NBJ5	GT251	Procollagen galactosyltransferase 1	N.D.	3.92 ± 0.12	0.000	-	yes

O00469	PLOD2	Procollagen-lysine,2-oxoglutarate 5-dioxygenase 2	N.D.	1.54 ± 0.03	0.000	-	yes
Q7Z3U7	MON2	Protein MON2 homolog	N.D.	1.87 ± 0.31	0.004	-	yes
Q15437	SC23B	Protein transport protein Sec23B	N.D.	4.07 ± 0.70	0.004	-	yes
Q9BWF3	RBM4	RNA-binding protein 4	N.D.	1.71 ± 0.20	0.001	-	yes
K7ERG4	K7ERG4	Small nuclear ribonucleoprotein Sm D2	N.D.	4.61 ± 0.26	0.000	-	yes

[1] From three independent experiments; [2] Based on two-tailed unpaired Student's *t* test t; [3] The *O*-GlcNAc Database v1.2 (https://www.oglcnac.mcw.edu/, accessed on 14 Dec 2021). ↓ means down-regulated; ↑ means up-regulated.

Table S2. Differential WGA-bound glycoproteins identified in cytosolic extracts from lung adenocarinoma cell lines CL1-1 and CL1-5.

Protein ID	Symbol	Protein Name	Normalized Spectral Counts (Mean ± SE) [1]		*p*-Value [2]	Fold Change	Listed in the O-GlcNAc Database [3]
			In CL1-1	In CL1-5			
		Down-regulated in CL1-5					
O14964	HGS	Hepatocyte growth factor-regulated tyrosine kinase substrate	10.54 ± 1.27	0.93 ± 0.93	0.004	↓11.32	yes
P46781	RS9	40S ribosomal protein S9	5.91 ± 1.33	0.74 ± 0.74	0.028	↓7.95	yes
P22059	OSBP1	Oxysterol-binding protein 1	3.43 ± 0.69	0.60 ± 0.60	0.036	↓5.71	yes
Q04637	IF4G1	Eukaryotic translation initiation factor 4 gamma 1	7.47 ± 1.35	1.34 ± 0.68	0.016	↓5.56	yes
Q06203	PUR1	Amidophosphoribosyltransferase	14.36 ± 0.83	2.72 ± 0.04	0.000	↓5.27	yes
P35580	MYH10	Myosin-10	46.35 ± 5.93	9.11 ± 1.59	0.004	↓5.08	yes
V9HW77	V9HW77	Epididymis luminal protein 211	27.73 ± 1.42	5.92 ± 1.03	0.000	↓4.68	no
Q9UNF0	PACN2	Protein kinase C and casein kinase substrate in neurons protein 2	8.50 ± 1.70	1.82 ± 1.04	0.028	↓4.67	yes
P54725	RD23A	UV excision repair protein RAD23 homolog A	3.46 ± 0.63	0.74 ± 0.74	0.050	↓4.65	yes
Q15631	TSN	Translin	5.27 ± 0.56	1.23 ± 0.68	0.010	↓4.30	yes
P48729	KC1A	Casein kinase I isoform alpha	2.50 ± 0.21	0.60 ± 0.60	0.040	↓4.17	yes
Q06830	PRDX1	Peroxiredoxin-1	5.28 ± 0.98	1.37 ± 0.70	0.031	↓3.85	yes
Q7L576	CYFP1	Cytoplasmic FMR1-interacting protein 1	14.82 ± 0.84	4.23 ± 0.59	0.001	↓3.50	yes
P49588	SYAC	Alanine--tRNA ligase, cytoplasmic	24.93 ± 2.30	7.29 ± 1.87	0.004	↓3.42	yes
P07195	LDHB	L-lactate dehydrogenase B chain	25.09 ± 3.18	7.72 ± 0.10	0.006	↓3.25	yes
P53396	ACLY	ATP-citrate synthase	16.62 ± 2.49	5.14 ± 0.24	0.010	↓3.24	yes
Q15435	PP1R7	Protein phosphatase 1 regulatory subunit 7	18.40 ± 1.00	5.76 ± 0.47	0.000	↓3.20	yes
Q9HB07	MYG1	UPF0160 protein MYG1, mitochondrial	4.83 ± 0.04	1.52 ± 0.80	0.014	↓3.17	yes
Q9NQW7	XPP1	Xaa-Pro aminopeptidase 1	6.98 ± 0.54	2.27 ± 1.19	0.022	↓3.08	yes
Q9UKK9	NUDT5	ADP-sugar pyrophosphatase	7.31 ± 0.13	2.42 ± 0.54	0.001	↓3.02	yes
B7Z2Z8	B7Z2Z8	T-complex protein 1 subunit delta	4.69 ± 0.39	1.64 ± 0.91	0.036	↓2.86	yes
P14868	SYDC	Aspartate--tRNA ligase, cytoplasmic	11.51 ± 0.76	4.07 ± 0.65	0.002	↓2.83	yes
P06132	DCUP	Uroporphyrinogen decarboxylase	5.60 ± 0.66	1.99 ± 1.01	0.040	↓2.82	yes
P55884	EIF3B	Eukaryotic translation initiation factor 3 subunit B	27.73 ± 1.58	10.29 ± 0.42	0.000	↓2.70	yes
Q9UNH7	SNX6	Sorting nexin-6	7.60 ± 0.67	2.89 ± 0.58	0.006	↓2.63	yes
P06753	TPM3	Tropomyosin alpha-3 chain	43.53 ± 1.70	16.91 ± 1.82	0.000	↓2.58	yes
P41250	SYG	Glycine--tRNA ligase	12.86 ± 2.08	5.01 ± 0.59	0.022	↓2.57	yes
E7EVA0	E7EVA0	Microtubule-associated protein	26.98 ± 1.06	10.71 ± 1.32	0.001	↓2.52	no

P67936	TPM4	Tropomyosin alpha-4 chain	57.10 ± 5.54	22.91 ± 3.63	0.007	↓2.49	yes
P12956	XRCC6	X-ray repair cross-complementing protein 6	14.12 ± 2.29	6.07 ± 0.74	0.029	↓2.33	yes
P21281	VATB2	V-type proton ATPase subunit B, brain isoform	6.72 ± 0.48	3.02 ± 0.36	0.004	↓2.23	yes
P61247	RS3A	40S ribosomal protein S3a	12.89 ± 1.12	5.88 ± 0.88	0.008	↓2.19	yes
Q7KZF4	SND1	Staphylococcal nuclease domain-containing protein 1	32.36 ± 2.62	14.81 ± 1.82	0.005	↓2.19	yes
A0MZ66	SHOT1	Shootin-1	14.06 ± 0.89	6.51 ± 0.62	0.002	↓2.16	yes
P51858	HDGF	Hepatoma-derived growth factor	21.46 ± 1.67	10.16 ± 1.30	0.006	↓2.11	yes
O00273	DFFA	DNA fragmentation factor subunit alpha	5.74 ± 0.46	2.73 ± 0.56	0.014	↓2.10	yes
P40123	CAP2	Adenylyl cyclase-associated protein 2	5.60 ± 0.19	2.72 ± 0.04	0.000	↓2.06	yes
Q15436	SC23A	Protein transport protein Sec23A	11.61 ± 1.66	5.76 ± 0.38	0.027	↓2.02	yes
P35749	MYH11	Myosin-11	8.44 ± 0.70	4.23 ± 0.59	0.010	↓2.00	yes
O76003	GLRX3	Glutaredoxin-3	7.74 ± 1.18	3.94 ± 0.18	0.033	↓1.97	yes
Q15046	SYK	Lysine: tRNA ligase	12.15 ± 0.23	6.33 ± 1.14	0.008	↓1.92	yes
Q9Y5X3	SNX5	Sorting nexin-5	6.06 ± 0.30	3.18 ± 0.26	0.002	↓1.91	yes
A0A024R9E2	A0A024R9E2	Poly(A) binding protein, cytoplasmic 1, isoform CRA_c	6.20 ± 0.69	3.31 ± 0.76	0.048	↓1.87	no
P49327	FAS	Fatty acid synthase	73.44 ± 6.17	39.26 ± 2.57	0.007	↓1.87	yes
P68104	EF1A1	Elongation factor 1-alpha 1	8.09 ± 0.20	4.36 ± 1.27	0.044	↓1.86	yes
P62140	PP1B	Serine/threonine-protein phosphatase PP1-beta catalytic subunit	7.77 ± 0.35	4.23 ± 0.90	0.021	↓1.84	yes
P07951	TPM2	Tropomyosin beta chain	64.51 ± 5.63	35.72 ± 8.39	0.047	↓1.81	yes
P07437	TBB5	Tubulin beta chain	14.93 ± 1.53	8.46 ± 0.46	0.016	↓1.76	yes
Q15691	MARE1	Microtubule-associated protein RP/EB family member 1	18.32 ± 1.41	10.43 ± 2.05	0.034	↓1.76	yes
Q9NYU2	UGGG1	UDP-glucose:glycoprotein glucosyltransferase 1	28.08 ± 1.47	16.24 ± 1.97	0.009	↓1.73	yes
O94979	SC31A	Protein transport protein Sec31A	13.94 ± 1.47	8.16 ± 0.61	0.022	↓1.71	yes
Q96QK1	VPS35	Vacuolar protein sorting-associated protein 35	9.19 ± 0.62	5.43 ± 0.72	0.017	↓1.69	yes
P26640	SYVC	Valine--tRNA ligase	22.07 ± 0.93	13.12 ± 1.67	0.009	↓1.68	yes
Q13347	EIF3I	Eukaryotic translation initiation factor 3 subunit I	6.35 ± 0.65	3.78 ± 0.14	0.018	↓1.68	yes
P12004	PCNA	Proliferating cell nuclear antigen	6.83 ± 0.52	4.08 ± 0.42	0.015	↓1.68	yes
Q02790	FKBP4	Peptidyl-prolyl cis-trans isomerase FKBP4	29.12 ± 2.38	17.65 ± 2.03	0.021	↓1.65	yes
P62753	RS6	40S ribosomal protein S6	6.70 ± 0.69	4.09 ± 0.31	0.026	↓1.64	yes
P33176	KINH	Kinesin-1 heavy chain	16.50 ± 0.59	10.10 ± 1.49	0.016	↓1.63	yes
Q14697	GANAB	Neutral alpha-glucosidase AB	46.40 ± 3.53	28.95 ± 2.14	0.013	↓1.60	yes
Q8N1G4	LRC47	Leucine-rich repeat-containing protein 47	7.17 ± 0.32	4.53 ± 0.22	0.003	↓1.58	yes
Q92526	TCPW	T-complex protein 1 subunit zeta-2	6.70 ± 0.05	4.24 ± 0.21	0.000	↓1.58	no
P09493	TPM1	Tropomyosin alpha-1 chain	29.14 ± 2.76	18.46 ± 0.83	0.021	↓1.58	yes
P38606	VATA	V-type proton ATPase catalytic subunit A	10.43 ± 0.49	6.80 ± 0.44	0.005	↓1.54	yes
P13639	EF2	Elongation factor 2	42.31 ± 2.06	27.94 ± 1.89	0.007	↓1.51	yes
P08195	4F2	4F2 cell-surface antigen heavy chain	4.72 ± 0.92	N.D.	0.007	-	yes
P62906	RL10A	60S ribosomal protein L10a	2.66 ± 0.22	N.D.	0.000	-	yes
P36578	RL4	60S ribosomal protein L4	4.97 ± 0.55	N.D.	0.001	-	yes
P62424	RL7A	60S ribosomal protein L7a	2.47 ± 0.62	N.D.	0.016	-	yes
Q13085	ACACA	Acetyl-CoA carboxylase 1	3.28 ± 0.74	N.D.	0.011	-	yes
P04083	ANXA1	Annexin A1	8.70 ± 0.63	N.D.	0.000	-	yes
P13861	KAP2	cAMP-dependent protein kinase type II-alpha regulatory subunit	2.34 ± 0.268	N.D.	0.001	-	yes

P30622	CLIP1	CAP-Gly domain-containing linker protein 1	2.35 ± 0.57	N.D.	0.015	-	yes
Q6P1N0	C2D1A	Coiled-coil and C2 domain-containing protein 1A	2.8 ± 0.46	N.D.	0.004	-	yes
P12532	KCRU	Creatine kinase U-type, mitochondrial	5.15 ± 1.01	N.D.	0.007	-	yes
Q13616	CUL1	Cullin-1	2.49 ± 0.09	N.D.	0.000	-	yes
Q86VP6	CAND1	Cullin-associated NEDD8-dissociated protein 1	2.92 ± 0.78	N.D.	0.020	-	yes
P15311	EZRI	Ezrin	3.45 ± 0.39	N.D.	0.001	-	yes
Q00839	HNRPU	Heterogeneous nuclear ribonucleoprotein U	2.98 ± 0.48	N.D.	0.004	-	yes
Q9Y2A7	NCKP1	Nck-associated protein 1	7.80 ± 0.48	N.D.	0.000	-	yes
O75694	NU155	Nuclear pore complex protein Nup155	1.56 ± 0.17	N.D.	0.001	-	yes
F8VR77	F8VR77	Proliferation-associated protein 2G4	4.53 ± 0.36	N.D.	0.000	-	yes
Q92734	TFG	Protein TFG	3.12 ± 0.16	N.D.	0.000	-	yes
Q8N1K5	THMS1	Protein THEMIS	1.88 ± 0.29	N.D.	0.003	-	yes
P35241	RADI	Radixin	4.98 ± 0.35	N.D.	0.000	-	yes
Q13464	ROCK1	Rho-associated protein kinase 1	3.28 ± 0.31	N.D.	0.001	-	yes
Q6IPH7	Q6IPH7	RPL14 protein	2.03 ± 0.20	N.D.	0.001	-	no
Q9Y6Y8	S23IP	SEC23-interacting protein	2.35 ± 0.33	N.D.	0.002	-	yes
P63151	2ABA	Serine/threonine-protein phosphatase 2A 55 kDa regulatory subunit B alpha isoform	2.83 ± 0.53	N.D.	0.006	-	yes
Q92783	STAM1	Signal transducing adapter molecule 1	4.52 ± 0.45	N.D.	0.001	-	yes
A0A0A0MTS7	A0A0A0MTS7	Titin	9.24 ± 1.45	N.D.	0.003	-	yes
P02786	TFR1	Transferrin receptor protein 1	4.73 ± 1.03	N.D.	0.010	-	yes
P29144	TPP2	Tripeptidyl-peptidase 2	6.07 ± 0.92	N.D.	0.003	-	yes
Q8TDJ5	Q8TDJ5	Tyrosine-protein kinase receptor	3.12 ± 0.16	N.D.	0.000	-	no
P54577	SYYC	Tyrosine--tRNA ligase, cytoplasmic	7.55 ± 1.98	N.D.	0.019	-	yes
O95155	UBE4B	Ubiquitin conjugation factor E4 B	2.77 ± 0.63	N.D.	0.012	-	yes
Up-regulated in CL1-5							
Q13813	SPTN1	Spectrin alpha chain, non-erythrocytic 1	3.54 ± 2.27	40.72 ± 6.90	0.007	↑11.52	yes
Q9H2G2	SLK	STE20-like serine/threonine-protein kinase	0.89 ± 0.89	8.94 ± 0.51	0.001	↑10.06	yes
P35609	ACTN2	Alpha-actinin-2	1.62 ± 1.62	12.83 ± 1.03	0.004	↑7.94	yes
O43707	ACTN4	Alpha-actinin-4	15.91 ± 0.77	95.46 ± 1.67	0.000	↑6.00	yes
Q5T8M8	Q5T8M8	Actin, alpha skeletal muscle	0.74 ± 0.74	3.95 ± 0.82	0.044	↑5.34	yes
P11310	ACADM	Medium-chain specific acyl-CoA dehydrogenase, mitochondrial	0.47 ± 0.47	2.42 ± 0.40	0.035	↑5.11	yes
A0A024R7P5	A0A024R7P5	Similar to Laminin receptor 1, isoform CRA_a	0.95 ± 0.95	4.84 ± 0.54	0.023	↑5.10	no
O75369	FLNB	Filamin-B	32.32 ± 1.73	148.56 ± 12.81	0.001	↑4.60	yes
A0A087X111	A0A087X111	G antigen 13	2.01 ± 0.36	7.57 ± 0.21	0.000	↑3.77	no
P06396	GELS	Gelsolin	5.61 ± 0.73	19.88 ± 2.64	0.007	↑3.54	yes
H0YN18	H0YN18	Proteasome subunit alpha type-4	2.23 ± 1.56	7.74 ± 0.80	0.035	↑3.48	yes
P08758	ANXA5	Annexin A5	2.60 ± 1.30	7.86 ± 0.60	0.021	↑3.02	yes
Q08043	ACTN3	Alpha-actinin-3	2.95 ± 1.50	7.85 ± 0.45	0.035	↑2.66	yes
P11413	G6PD	Glucose-6-phosphate 1-dehydrogenase	1.55 ± 0.78	4.08 ± 0.23	0.035	↑2.64	yes
P12814	ACTN1	Alpha-actinin-1	18.09 ± 2.47	47.35 ± 1.28	0.001	↑2.62	yes
O00232	PSD12	26S proteasome non-ATPase regulatory subunit 12	2.98 ± 0.38	6.36 ± 0.54	0.007	↑2.13	yes
Q16576	RBBP7	Histone-binding protein RBBP7	3.75 ± 0.51	7.59 ± 0.91	0.021	↑2.02	yes

O75131	CPNE3	Copine-3	5.66 ± 1.17	11.36 ± 0.41	0.010	↑2.01	yes
P45974	UBP5	Ubiquitin carboxyl-terminal hydrolase 5	8.42 ± 0.47	15.88 ± 0.09	0.000	↑1.89	yes
P56537	IF6	Eukaryotic translation initiation factor 6	2.80 ± 0.22	5.16 ± 0.80	0.046	↑1.84	yes
P08107	HSP71	70-kDa Heat-shock protein 1A/1B	33.88 ± 1.91	55.01 ± 3.94	0.009	↑1.62	no
Q16531	DDB1	DNA damage-binding protein 1	32.49 ± 2.12	50.70 ± 0.97	0.002	↑1.56	yes
P35611	ADDA	Alpha-adducin	N.D.	5.58 ± 1.00	0.005	-	yes
P06733	ENOA	Alpha-enolase	N.D.	3.34 ± 0.57	0.004	-	yes
Q8WWH9	Q8WWH9	Amphiphysin IIb-1	N.D.	2.85 ± 0.77	0.021	-	no
P20810	ICAL	Calpastatin	N.D.	3.65 ± 0.73	0.008	-	yes
Q13185	CBX3	Chromobox protein homolog 3	N.D.	1.82 ± 0.26	0.002	-	yes
P21333	FLNA	Filamin-A	N.D.	29.23 ± 1.24	0.000	-	yes
P09104	ENOG	Gamma-enolase	N.D.	3.79 ± 0.45	0.001	-	yes
P05556	ITB1	Integrin beta-1	N.D.	4.83 ± 0.34	0.000	-	yes
P46821	MAP1B	Microtubule-associated protein 1B	N.D.	2.57 ± 0.65	0.017	-	yes
O00499	BIN1	Myc box-dependent-interacting protein 1	N.D.	2.85 ± 0.77	0.021	-	yes
Q9UJ70	NAGK	N-acetyl-D-glucosamine kinase	N.D.	5.17 ± 1.25	0.014	-	yes
Q96TA1	NIBL1	Niban-like protein 1	N.D.	8.33 ± 1.09	0.002	-	yes
P0CG47	UBB	Polyubiquitin-B	N.D.	3.35 ± 0.66	0.007	-	yes
P0CG48	UBC	Polyubiquitin-C	N.D.	3.35 ± 0.66	0.007	-	yes
O15460	P4HA2	Prolyl 4-hydroxylase subunit alpha-2	N.D.	3.78 ± 0.14	0.000	-	yes
P62714	PP2AB	Serine/threonine-protein phosphatase 2A catalytic subunit beta isoform	N.D.	2.42 ± 0.31	0.002	-	yes
Q08209	PP2BA	Serine/threonine-protein phosphatase 2B catalytic subunit alpha isoform	N.D.	2.43 ± 0.19	0.000	-	yes
Q01082	SPTB2	Spectrin beta chain, non-erythrocytic 1	N.D.	36.26 ± 4.34	0.001	-	yes
Q9H254	SPTN4	Spectrin beta chain, non-erythrocytic 4	N.D.	3.17 ± 0.23	0.000	-	yes
P62979	RS27A	Ubiquitin-40S ribosomal protein S27a	N.D.	3.35 ± 0.66	0.007	-	yes
P62987	RL40	Ubiquitin-60S ribosomal protein L40	N.D.	3.35 ± 0.66	0.007	-	yes
P08670	VIME	Vimentin	N.D.	9.17 ± 2.27	0.016	-	yes

[1] From three independent experiments; [2] Based on two-tailed unpaired Student's *t* test; [3] The *O*-GlcNAc Database v1.2 (https://www.oglcnac.mcw.edu/, accessed on 14 Dec 2021. ↓ means down-regulated; ↑ means up-regulated.

Table S3. Significant network functions for differential WGA-bound glycoproteins identified in lung adenocarinoma cell lines CL1-1 and CL1-5.

No.	Associated Network Functions	Score	Molecules in the Network
1	Neurological Disease, Skeletal and Muscular Disorders, Hereditary Disorder,	59	26S Proteasome, Alpha tubulin, ANXA1, ANXA2, C12orf10, calpain, CCT4, COLGALT1, CPSF2, CUL1, CYFIP1, DARS, EEF1A1, GARS, HSPA4, HSPB1, KARS, MAP1B, MAPK, NPM1, NUP153, PLOD3, PSMA3, PSMD4, RAE1, Rnr, RPS9, RPS3A, RPSA, TSN, UBB, UBQLN1, UGGT1, VARS, VCP
2	Cellular Assembly, and Organization, Cellular Function	48	ACTG1, Actin, ACTN1, ACTN4, ADCY, ADRB, Alpha Actinin, Alpha catenin, ANXA6, API5, ATP6V1A, CaMKII, CAP2, CLIP1, ERK, EZR, FLNA, FLNB, IQGAP1, Lh, MAPRE1, MYH9, NASP, P4HA2, PLCB3, PLEC, PPP1CB, PRKAR2A, Spectrin, SPTAN1, SPTBN1, SPTBN4, THEMIS, TPM1, TUBB

	and Maintenance, Cell-To-Cell Signaling and Interaction		
3	Cell Morphology, Protein Trafficking, Cellular Assembly and Organization	42	19S proteasome, 20S proteasome, AOC1, APC/APC2, CPSF7, Dynamin, DYNC1I2, ERK1/2, HGS, Immunoproteasome Pa28/20s, PACSIN2, Proteasome PA700/20s, PSMA, PSMA1, PSMA4, PSMA6, PSMB7, PSMD, PSMD12, PSMD14, PSME4, RAD23A, Sec23, SEC23A, SEC23B, SEC23IP, SEC31A, SNX5, SNX6, STAM, UBA1, UBE4B, Ubiquitin, USP5, VPS35
4	RNA Post-Transcriptional Modification, Cellular Assembly and Organization, Cellular Compromise	38	60S ribosomal subunit, ATPase, CAND1, EEF2, ENO1, Erm, FUS, HNRNPH3, HNRNPR, HNRNPU, ILF2, JINK1/2, Jnk, MACF1, MSH2, N-cor, P glycoprotein, PSMC4, PSMC5, Rar, RDX, Rho gdi, RPL4, RPL14, RPL10A, RPL7A, Rxr, SERBP1, SLK, T3-TR-RXR, TCERG1, TFIIH, thyroid hormone receptor, UBA52, XRN2
5	RNA Post-Transcriptional Modification, Cell Cycle, DNA Replication, Recombination, and Repair	33	ABCF1, Akt, Cdc2, Cyclin A, Cyclin D, Cyclin E, E2f, EIF3, EIF6, EIF3B, EIF3I, Eif4g, EIF4G1, G3BP1, Gcn5l, HNRNPC, LSM4, mediator, Mre11, PA2G4, PABPC1, PLRG1, PPP4R3A, PRPF19, Rb, Ribosomal 40S subunit, RPA, RPS6, RPS27A, SMC1A, SND1, snRNP, SNRNP40, SNRPD2, ZNF207

Table S4. IPA-identified significant canonical pathways associated with differential WGA-bound glycoproteins in lung adenocarinoma cell lines CL1-1 and CL1-5.

**Pathway Name**	***p*-Value**	**Ratio**
All differential WGA-bound glycoproteins identified from CL1-1 and CL1-5 cells		
Actin Cytoskeleton Signaling	5.91×10^{-15}	22/228 (9.6%)
Protein Ubiquitination Pathway	4.35×10^{-11}	19/255 (7.5%)
ILK Signaling	4.10×10^{-10}	16/196 (8.2%)
Remodeling of Epithelial Adherens Junctions	2.85×10^{-9}	10/68 (14.7%)
EIF2 Signaling	3.10×10^{-9}	15/194 (7.7%)
WGA-bound glycoproteins Down-regulated in CL1-5 cells		
Actin Cytoskeleton Signaling	1.49×10^{-12}	16/228 (7.0%)
EIF2 Signaling	4.44×10^{-9}	12/194 (6.2%)
Cellular Effects of Sildenafil (Viagra)	1.13×10^{-8}	10/130 (7.7%)
tRNA Charging	1.68×10^{-7}	6/39 (15.4%)
Regulation of eIF4 and p70S6K Signaling	7.77×10^{-7}	9/157 (5.7%)
WGA-bound glycoproteins Up-regulated in CL1-5 cells		
Protein Ubiquitination Pathway	9.75×10^{-12}	14/255 (5.5%)
ILK Signaling	2.13×10^{-8}	10/196 (5.1%)
Regulation of Cellular Mechanics by Calpain Protease	2.27×10^{-7}	6/57 (10.5%)
Sertoli Cell-Sertoli Cell Junction Signaling	1.70×10^{-5}	7/178 (3.9%)
Actin Cytoskeleton Signaling	8.24×10^{-5}	7/228 (3.1%)

Table S5. Statistically significant molecular and cellular functions associated with differential WGA-bound glycoproteins identified in lung adenocarinoma cell lines CL1-1 and CL1-5.

**Molecular and Cellular Functions**	**p-Value**	**Number of Molecules**
Differentially expressed WGA-bound glycoproteins identified from CL1-1 and CL1-5 cells		
Cell Death and Survival	4.83×10^{-3}–6.99×10^{-20}	121
Cellular Growth and Proliferation	4.72×10^{-3}–1.00×10^{-18}	138
Cellular Assembly and Organization	4.39×10^{-3}–5.46×10^{-12}	94
Cellular Function and Maintenance	4.44×10^{-3}–5.46×10^{-12}	101
Cellular Development	4.58×10^{-3}–5.24×10^{-11}	119
WGA-bound glycoproteins Down-regulated in CL1-5 cells		
Cell Death and Survival	6.39×10^{-3}–6.50×10^{-11}	68
Cellular Growth and Proliferation	6.39×10^{-3}–4.94×10^{-10}	79
Cellular Assembly and Organization	6.39×10^{-3}–5.64×10^{-10}	53
Cellular Function and Maintenance	6.39×10^{-3}–5.64×10^{-10}	66
Protein Synthesis	1.44×10^{-3}–1.11×10^{-8}	31
WGA-bound glycoproteins Up-regulated in CL1-5 cells		
Cell Death and Survival	6.28×10^{-3}–1.87×10^{-13}	57
Cellular Growth and Proliferation	5.22×10^{-3}–9.49×10^{-12}	63
RNA Post-Transcriptional Modification	2.07×10^{-3}–2.03×10^{-8}	12
Cellular Assembly and Organization	5.73×10^{-3}–1.29×10^{-7}	37
Cellular Development	5.29×10^{-3}–2.30×10^{-7}	56

Table S6. Association of SAM68 expression with lung adenocarcinoma stage.

SAM68 Expression	Tumor Stage		*p*-Value [1]
	Stage I	Stage II/III/IV	
Low SAM68 Count	58	31	-
% within Stage	59.2%	40.8%	-
High SAM68 Count	40	45	-
% within Stage	40.8%	59.2%	$p = 0.016$
Total Count	98	76	-
% within Stage	100.0%	100.0%	-

[1] Two-sided log-rank test.

© 2021 by the authors. Licensee MDPI, Basel, Switzerland. This article is an open access article distributed under the terms and conditions of the Creative Commons Attribution (CC BY) license (http://creativecommons.org/licenses/by/4.0/).
